# Supplementary material for: The use of brain functional magnetic resonance imaging to determine the mechanism of action of gabapentin in managing chronic pelvic pain in women: a pilot study
Source: BMJ Open. 2019 Jun 27;9(6):e026152. doi: 10.1136/bmjopen-2018-026152 (PMC6597644; doi:10.1136/bmjopen-2018-026152)
Supplement: Author's manuscript [file bmjopen-2018-026152.draft_revisions.pdf]

BMJ Open is committed to open peer review. As part of this commitment we make the peer review history of every article we publish publicly available.

When an article is published we post the peer reviewers' comments and the authors' responses online. We also post the versions of the paper that were used during peer review. These are the versions that the peer review comments apply to.

The versions of the paper that follow are the versions that were submitted during the peer review process. They are not the versions of record or the final published versions. They should not be cited or distributed as the published version of this manuscript.

BMJ Open is an open access journal and the full, final, typeset and author-corrected version of record of the manuscript is available on our site with no access controls, subscription charges or pay-per-view fees (http://bmjopen.bmj.com).

If you have any questions on BMJ Open's open peer review process please email info.bmjopen@bmj.com

BMJ Open

The use of brain functional magnetic resonance imaging to determine the mechanism of action of gabapentin in managing chronic pelvic pain in women: a pilot study

Journal:	*BMJ Open*
Manuscript ID	bmjopen-2018-026152
Article Type:	Research
Date Submitted by the Author:	19-Aug-2018
Complete List of Authors:	Seretny, Marta; University of Edinburgh, Edinburgh Cancer Research UK Centre<br>Murray, Sarah; University of Edinburgh, MRC Centre for Reproductive Health<br>Whitaker, Lucy; University of Edinburgh, MRC Centre for Reproductive Health<br>Murnane, Jonathan; University of Edinburgh, Queens Medical Research Institute and Edinburgh Imaging facility (QMRI)<br>Pernet, Cyril; University of Edinburgh, Queens Medical Research Institute and Edinburgh Imaging facility (QMRI)<br>Whalley, Heather; University of Edinburgh, Centre for Clinical Brain Sciences Centre for Genomic and Experimental Medicine<br>Horne, Andrew
Keywords:	Chronic pelvic pain, Functional Magnetic Resonance Imaging, Pilot Randomised Control Trial (RCT), Gabapentin, Placebo

SCHOLARONE™
Manuscripts

The use of brain functional magnetic resonance imaging to determine the mechanism of action of gabapentin in managing chronic pelvic pain in women: a pilot study

Authors

Marta Seretny MD, MPH, PhD, FRCA University of Edinburgh

Sarah Murray MB ChB, MRCOG University of Edinburgh

Lucy Whitaker MB ChB, MSc, MRCOG, MD University of Edinburgh

Jonathan Murnane BSc, MSc University of Edinburgh

Cyril R Pernet PhD University of Edinburgh

Heather C Whalley University of Edinburgh

*Andrew W Horne MB ChB, PhD, FRCOG University of Edinburgh

*Contact Details Corresponding Author:

Prof Andrew Horne

Email: Andrew.Horne@ed.ac.uk

Address: MRC Centre for Reproductive Health, The University of Edinburgh

Queen's Medical Research Institute

47 Little France Crescent

Edinburgh EH16 4TJ

Phone: +44 131 242 6162

Word Count: 2549

For peer review only

Article Summary

Strengths and Limitations of this study:

- This pilot study includes women with chronic pelvic pain (CPP) randomised to receive placebo or gabapentin for its management.
- All participants in this pilot study underwent a brain fMRI scan.
- This study estimated sample size for a larger fMRI study assessing gabapentin in CPP and also assessed study acceptability.
- The fMRI sample was too small to draw any statistical conclusions, all results are suggestive of trends only.

For peer review only

Key Words:

Chronic Pelvic Pain, Functional Magnetic Resonance Imaging (fMRI), Gabapentin, Placebo, Pilot Randomised Control Trial (RCT), Pilot Study

Abstract

Objective: To inform feasibility and design of a future randomised controlled trial (RCT) using brain functional magnetic resonance imaging (fMRI) to determine the mechanism of action of gabapentin in managing chronic pelvic pain (CPP) in women.

Design: Mechanistic study embedded in pilot RCT.

Setting: University Hospital

Participants: Twelve women (18–50 years) with CPP and no pelvic pathology (follow up completed March 2014).

Intervention: Oral gabapentin (300-2700mg) or matched placebo.

Outcome measures: After six weeks of treatment, participants underwent functional MRI (fMRI) of the brain (Verio Siemens 3T MRI) during which noxious heat and punctate stimuli were delivered to the pelvis and arm. Outcome measures included pain (visual analogue scale), Blood Oxygen Level Dependent (BOLD) signal change and a semi structured acceptability questionnaire at study completion prior to unblinding.

Results: Full datasets were obtained for 11 participants. Following noxious heat to the abdomen the gabapentin group (GG) had lower pain scores (Mean: 3.8 [SD 2.2]) than the placebo group (PG) (Mean: 5.8 [SD 0.9]). This was also the case for noxious heat to the arm with the GG having lower pain scores (Mean: 2.6 [SD 2.5]) then the PG (Mean: 6.2 [SD 1.1]). Activation in the left posterior insula was reduced in the gabapentin compared to the placebo group following punctate stimuli (1.4 95%CI: 0.17 to 2.5, p =0.015) and trended towards significance for noxious heat to the hand (3.03 95%CI: -0.8 to 6.8, p=0.051). Seven out of 12

For peer review only

participants completed the acceptability questionnaire. 71% (five out of seven) described their participation in the fMRI study as positive; the remaining two rated it as a negative experience.

Conclusions: Incorporating brain fMRI in a future RCT to determine the mechanism of action of gabapentin in managing CPP in women was feasible and acceptable to most women.

Trial Registration: ISRCTN70960777.

For peer review only

Introduction

Chronic pelvic pain (CPP) affects >3.3 billion women worldwide and costs an estimated >$225 million/year (£150 million) in socioeconomic costs in the UK alone[1]. Underlying pathology, such as endometriosis or adhesions, can be identified in around 65% of women with CPP. In the remaining 35% of women, pain symptoms have no clear aetiology[2, 3]. It is increasingly recognised that women with CPP of unknown aetiology likely represent a subgroup of patients who have undergone central sensitisation (CS), an increased responsiveness of central nociceptive neurons to their normal or sub-threshold afferent input[4]. The treatment for women with CPP of unknown aetiology is challenging[5].

Currently there are no evidence-based analgesics for CPP[6] but gabapentin (a GABA analogue) is increasingly prescribed due to its known effectiveness in other neuropathic pain states[6, 7]. The true efficacy, mechanism of action and side effect profile of gabapentin in women with CPP remains unknown. However, in general investigation of analgesic drug efficacy in clinical trials is not straightforward. Due to the subjective nature of pain and the influence of an active physiological placebo response, trials investigating analgesics, even when appropriately powered and carefully executed, often produce small effect sizes that are difficult to interpret clinically [8]. Nevertheless, the increase in the use of adjuvant research tools, such as brain functional magnetic resonance imaging (fMRI), has been shown to strengthen and mechanistically explain findings[9].

For peer review only

We therefore undertook a pilot exploratory study to determine the feasibility of investigating the mechanism of action of gabapentin in managing women with CPP using brain fMRI. The objectives of the study were to:

1) To determine whether it is possible to recruit women with CPP, who had agreed to participate in a related pilot clinical trial and received gabapentin or placebo, to partake in an embedded mechanistic study and undergo a brain fMRI scan.

2) To analyze the fMRI brain data and use it for sample size calculation for a future large fMRI brain study investigating the mechanism of action of gabapentin in managing CPP in women.

3) To assess if the fMRI and associated noxious stimuli were acceptable to participants.

For peer review only

Materials and Methods

Study design

This was a pilot exploratory mechanistic study. The flow of patients in the study is detailed in a CONSORT diagram (see figure 1).

Setting

Women with CPP in NHS Lothian (UK), already recruited as part of a pilot RCT conducted in NHS Lothian and NHS Grampian (UK) to investigate the efficacy of gabapentin versus placebo in CPP management[10, 11], were approached and invited to participate in this embedded fMRI substudy. The brain fMRI scans were carried out on a Siemens Verio 3T MRI Scanner located at the Edinburgh Imaging Facility, Queens Medical Research Institute (previously known as the Edinburgh Clinical Research Imaging Centre), at the University of Edinburgh (UK). Other study measures were tested in NHS Lothian clinical research facilities or, where appropriate, via the telephone.

Inclusion criteria

- Women aged between 18 and 50 years
- Chronic pelvic pain (non-cyclical pain with or without dysmenorrhoea or dyspareunia) located within the true pelvis or between and below anterior iliac crests
- Associated functional disability
- No obvious pelvic pathology at laparoscopy (<36 months and >2 weeks ago)

For peer review only

- Normal pelvic ultrasound (<36 months)
- Using effective contraception e.g. barrier methods, combined oral contraceptive pill, etc.
- Written informed consent

Exclusion criteria

- Known pelvic pathology (for example, endometriosis, cyst, fibroid>3cm)
- Past history of having taken gabapentin or pregabalin
- Due to undergo surgery in the next six months
- History of significant renal impairment
- Allergic to gabapentin or excipients
- Breast feeding
- Pregnancy or planning pregnancy in the next six months
- Metal implant/pacemaker/claustrophobia (fMRI subgroup).

Intervention

Patients were randomised to receive gabapentin at an increasing dose (300-2700mg) or identical placebo tablet. Full details are published in the related clinical trial protocol and results[10 11].

Outcome Measures

Women underwent an fMRI scan of the brain after six weeks of treatment. The following sequences were collected: structural MPRAGE sequence, resting state BOLD sequence where participants where asked to fixate on a cross, BOLD sequences during which punctate and noxious heat stimuli were applied to the

For peer review only

arm and lower abdomen respectively. The order of the latter was randomised (some participants had thermal stimulation applied to the arm then abdomen, whilst others had it applied in the reverse order). Thermal stimulus was individually thresholded outside the scanner for each participant. For the arm, a *MEDOC Pathway* thermode was placed on the inner arm, three fingerbreadths proximal to the wrist joint. Temperature was increased from 30C until the patient reported a pain score of five out of ten. The same was repeated for the lower abdomen (three finger breadths above the pubic bone) until the patient reported a pain score of five out of ten. These temperatures were noted and used in the scanner in a block experimental design; temperature at neutral for 10seconds and then increased to the participant's pre-established noxious heat threshold and held for 15seconds and then returned to neutral). The duration of the thermal blocks was ten minutes on the arm and ten minutes on the lower abdomen. Pain scores were recorded in real time during the application of the thermal stimulus using a visual analogue scale presented to participants on a screen inside the scanner. Each participant used an automated pointer to rate her pain. Standardised punctate stimuli were applied to the lower abdomen, three fingerbreadths above the pubic bone using a 300g *Touch Test™* von Frey filament (6.65mm) in an event related experimental design. The full duration of the punctate experiment was ten minutes. To assess the acceptability of the fMRI substudy, a randomly selected subset of women, were invited to complete a semi-structured questionnaire administered by telephone at the conclusion of the study protocol prior to unblinding. The questions were designed to assess the participant's feelings and experiences related to the fMRI scan (table 1). The responses were recorded on a structured coding sheet. Respondents were asked

For peer review only

to answer each question using a five-point positive (one) to negative (five) scale, with the option of adding free comments.

Regarding the fMRI substudy	Positive				Negative
	1	2	3	4	5
A. … was the choice to opt-in or out a positive or negative one? Did you feel pressured?					
B. … did you feel you were fully informed as to why you were having it?					
C. … was the fMRI a comfortable experience?					
D. … how did you find having the blood sample taken?					
E. … experiences and thoughts on the pain threshold exam					

Table 1. Semi Structured Study Acceptability Questionnaire. Women were asked these questions during a phone interview-taking place prior to study unblinding. The researcher recorded free comments elaborating each question.

Statistical Analysis

All statistical analysis was carried out prior to participant and clinician unblinding. FMRI statistical analysis was carried out using *Statistical Parametric Mapping* (SPM8) software. After standard pre processing steps (slice time correction, realignment, coregistartion, normalisation and smoothing), first level within subject analysis was performed to obtain an individual response to each type of noxious stimuli. Following this, group level whole brain (z threshold =2.3, regions considered significant at p <0.05, false discovery rate cluster corrected – p=0.001 cluster forming threshold) and region of interest (ROI) analysis were carried out. ROI's chosen *a priori* were anterior (AI) and posterior (PI) insula bilaterally, and periaqueductal grey (PAG); regions hypothesised to be important in the mechanism of action of gabapentin[12]. Stimulus-related activity was

For peer review only

extracted from ROIs for each subject and compared using independent sample, one-tailed t tests. Mean BOLD signal differences with 95% confidence interval (CI) are reported. Sample size calculations for a planned future mechanistic study investigating the mechanism of action of gabapentin versus placebo to manage CPP using fMRI, were also carried out. Non-fMRI data was analysed using *IBM SPSS Statistics for Macintosh, Version 22.0* software. Where appropriate, continuous variable were compared using an independent t test while categorical variables were compared using a chi-squared test.

Qualitative Analysis

Questionnaire data was assessed thematically in order to identify participant's feelings and experiences related to the fMRI scan and associated noxious heat thresholding. Free comments were reviewed to gain greater understanding on themes important to individual women in the study.

Ethical Approval

The Scotland A Research Ethics committee gave approval for this study (REC 12/SS/0005) on November 13th 2012. All participants gave written informed consent prior to taking part in this study. Consent was also reconfirmed verbally before every fMRI scan.

Patient and Public Involvement

The patient co-founder of the Pelvic Pain Support Network helped with the design of this study and also assisted with gathering feedback regarding the

For peer review only - http://bmjopen.bmj.com/site/about/guidelines.xhtml

proposed study within the charity. A short description of the proposed pilot study, planned resulting RCT and a series of related questions were distributed amongst members. Encouragingly, from a survey of 85 women involved in the charity, 69% responds said that they would consider taking part in this pilot.

Results

Recruitment

137 women fulfilled the eligibility criteria for the main pilot clinical trial, and 47 women were randomised to treatment[11]. Of the 47 women who were randomised, 12 participants were randomly approached to take part in the fMRI sub-study. All of the women approached consented to undergo a brain fMRI scan, completing the full scanning protocol. Their baseline demographics are shown in table 2. One subject (on placebo) was excluded from analysis following discovery of corrupted scan data at the analysis stage of the study.

**Characteristic**	**Gabapentin (% of 6 or 95% CI)**	**Placebo (% of 6 or 95% CI)**
Age (years)	31.7 (20.3 – 43.0)	30.3 (23.1 – 37.5)
Pain Score (0-10)	6.6 (5.8 – 7.5)	5.5 (3.3– 7.7)
BMI (kg/m^2)	25.05 (21.0 – 29.1)	26.05 (21.2– 30.9)
HADS score (max score 21)	9.7 (6.6 – 12.7)	8 (3.4 -12.5)
Parity:<br>Nulliparous<br>Parous	 4 (66.7%)<br>2 (33.3%)	 4(66.7%))<br>2(33.3%))
Higher Education Received:<br>Highschool<br>College/University	 0(0%)<br>6(100%)	 1(16.7%)<br>5(83.3%)

Table 2. Demographics of women who underwent fMRI brain. HADS= hospital anxiety and depression score.

fMRI Analysis

fMRI brain scan data for six subjects receiving gabapentin and five receiving placebo was analysed. Average pain ratings following noxious stimuli of the abdomen were lower for the gabapentin group (Mean: 3.8 [SD 2.2]) than the placebo group (Mean: 5.8 [SD 0.9]). This was also the case for noxious heat applied to the arm where the gabapentin group had lower average pain scores (Mean: 2.6 [SD 2.5]) than the placebo group (Mean: 6.2 [SD 1.1]). As expected, due to the exploratory sample size, group level whole brain analysis did not yield statistically significant differences. In terms of ROI analysis (figure 2); activation in the left PI was significantly reduced in the gabapentin compared to the placebo groups following punctate stimuli (1.4 95%CI: 0.17 to 2.5, p =0.015) and trended towards significance for noxious heat applied to the hand (3.03 95%CI: -0.8 to 6.8, p=0.051). Although not statistically significant, activation in the remaining ROIs was reduced in the gabapentin group compared to placebo. A sample size calculation from these results, suggest that for a mechanistic sub study embedded in a larger double blind RCT investigating the efficacy of gabapentin versus placebo in CPP, 15 women per group (a total of 30 brain fMRI scans) are required for 80% power and p=0.05 significance level, sampling ratio 1:1, with two independent samples and two-sided testing. This was based on a 1.4% difference in BOLD signal in the LPI in response to thermal stimulation of the hand and punctate stimulation of the abdomen.

For peer review only

FMRI Substudy Acceptability

Seven women were randomly invited to, and completed the telephone administered semi- structured acceptability questionnaire. Five women rated the fMRI as a positive experience, scoring it as 1 or 2 on a 1 - 5 point, positive to negative scale. Two participants scored their experience as 'four' on the scale. One of these commented that the trial substudy process was not explained clearly enough. This participant was also disappointed by lack of feedback after the scan. The other participant rating the experience negatively felt that an in-person explanation rather than the information leaflet would have been clearer. All participants felt the option of opting in or out of the mechanistic fMRI substudy was positive. The majority of women found the experience of the actual scan positive or neutral, with similar ratings given to the quality of information given before the scan, as well as their experience of the noxious pain thresholding. One participant noted that prior to the study she had anxiety related to MRI scans and that study participation actually helped her overcome her fear of MRI. Only one participant felt that the information given before the scan, the actual scan experience, as well as the noxious thresholding was a negative experience for her (a score of four on the five-point scale).

Discussion

Recruitment

Our study met our objectives in relation to establishing feasibility of recruitment. All of the participants approached for brain fMRI consented to this part of the study and completed the full hour-long protocol in the scanner. Failure to recruit to trials is known to be a major limitation in clinical research, prompting new

For peer review only

recommendations aimed at improving approaches to recruitment[13]. Assessment of study feasibility is a part of this improvement process. Pilot studies are considered a subtype of formal feasibility assessments[14]. Further, pilot studies are deemed valuable in ascertaining unknown factors such as recruitment to novel outcome measures, such as in our case brain fMRI in CPP patients[15]. We feel our successful recruitment to this study supports future fMRI research investigating the mechanism of action of gabapentin for the management of women with CPP.

fMRI Analysis

The key outcome of our fMRI data analysis was calculation of sample size for a planned future brain fMRI study to investigate the mechanism of action of gabapentin in the management of women with CPP. Sample size calculations for fMRI studies have become a standard part of good MRI research methodology[16]. We also explored BOLD signal changes in response to punctate and noxious heat stimuli, in regions of the brain known to be involved in pain processing. We found a trend suggesting decreased pain processing in the posterior insula in women who were receiving gabapentin compared those who were receiving placebo. These findings fit with what is known about the fundamental role of the insula in central sensitisation and pain processing[17, 18]. Specifically, in CPP this region is known to have altered structure and connectivity[19]. Moreover, central sensitisation is postulated to be an important mechanism in the maintenance of CPP[20]. Neuromodulators such as gabapentin have been shown in neuroimaging studies to modify central sensitisation pain states[12]. This would fit with our results suggesting decreased pain processing in the PI in women with CPP

For peer review only

receiving gabapentin, an area known to be dense in neurons containing GABAergic receptors. Further work in a larger mechanistic fMRI study is needed to confirm and elucidate these findings.

Study Acceptability

Patient engagement in clinical trials is known to improve study recruitment and retention[21]. Specific to pain research and analgesic RCTs, many issues limit patient participation in pain trials utilising fMRI, not least the actual experience of chronic pain[22]. Decreased participation of subsets of pain patients in pain medication trials can introduce potential biases into study findings [23]. Understanding patient experiences, which in our case helped highlight the need for clearer communication around study logistics, will help utilise fMRI in future mechanistic probing of gabapentin efficacy in CPP. This is particularly valuable, as the utility of fMRI in chronic pain management research is deemed to have growing importance[24].

Study Strengths and Limitations

The major limitation of our fMRI data analysis lies in our small sample size. Approaches to brain fMRI analysis are varied and maybe prone to false positive or non-reproducible results[25]. This is particularly true when small datasets are used [26]. It is therefore possible that a different approach to the analysis of our data may have yielded different results. We therefore interpret these findings as interesting tends, in need of further validation, and we have made our fMRI dataset available to interested researchers who may wish to re analyse our data or combine it with another relevant dataset. The strengths of this study relate to

For peer review only

all the study aims being fulfilled and clearly reported. This study is one of the first to utilise fMRI in the assessment of women with CPP receiving gabapentin or placebo.

Conclusion

We have demonstrated that it is possible to recruit women with CPP participating in a pilot RCT of gabapentin versus placebo, to take part in a mechanistic substudy involving a brain fMRI scan. Most participants in our pilot found the MRI scan, including administration of noxious stimuli acceptable. BOLD signal changes identified in our analysis are likely useful guides for informing understanding of the mechanism of action of gabapentin in CPP. Sample size calculations resulting from this work have been used to inform the design of a mechanistic fMRI substudy in a larger RCT investigating gabapentin versus placebo for the management of CPP in women.

For peer review only

Figure Legends

Figure 1. CONSORT diagram

Figure 2. Group differences in BOLD Signal Change in regions of interest
PAG = periaqueductal grey. BOLD = blood oxygen level dependent. Although not statistically significant the analysis showed decreased activation for the gabapentin group in all ROI following all stimuli, except in the RAI and LPI following abdominal noxious heat. The Left PI showed significantly decreased mean activation (p=0.015 independent sample, one sided t-test) in response to punctate stimuli on the abdomen and tended towards significance (p=0.051) for punctate stimuli of the hand. Our results suggest that gabapentin changes the set point for acute pain modulation in CPP patients. The PI has been shown to have strong connectivity with the somatosensory cortex and is involved in transitioning from painful to non-painful sensations. Significantly decreased pain-related activity in this region in the gabapentin group highlights the potential central efficacy of this drug in women with CPP.

For peer review only

Conflict of Interest

We have read and understood the BMJ Open policy on declaration of interests and declare that we have no competing interests

Author Statement

MS, AH, CP where involved in the design and set up of the study. MS, LH, SM, JM and CP were involved in scan data collection. CP analysed the fMRI data. HW performed the sample size calculation. MS analysed the raw BOLD signal ROI differences. All authors contributed to the writing of this manuscript.

Data Statement

All data from this study is currently held securely at the University of Edinburgh. Anonymised fMRI data is available to interested researchers and can be accessed at: https://datashare.is.ed.ac.uk/handle/10283/3153

Acknowledgments

We thank Katy Vincent (DPhil, BSc, MBBS, MRCOG) who was instrumental in guiding the set up and data analysis for this study. Thank you to Scott Semple (PhD) and Neil Roberts (PhD), MRI physicists for their assistance in setting up additional data acquisition equipment and monitoring in the MRI scanner. We would also like to thank Ms Jennifer Brawn (MSc, DPhil) for organising scan sequence transfer and set up between Oxford and Edinburgh. We thank all the radiographers at the Clinical Research Imaging Centre (CRIC) Edinburgh, for their contribution to data collection. We also thank Mrs Ann Doust and Mrs Helen Dewart for organising patient recruitment and follow up. We would like to

For peer review only

thank Judy Birch JB, the patient liaison and representative of the Pelvic Pain Support Network for her advice and input into this study. Finally, and perhaps most importantly we would like to thank all the women who took part in this study.

Funding

This work was supported by a grant from the Chief Scientist's Office Scotland (CZH/4/688) and an MRC Centre Grant Mr/N022556/1. MS was funded by the Wellcome Trust via the Scottish Translational Medicine and Therapeutics Initiative (STMTI). JM held a PhD studentship funded by the Mentholatum company. The funders did not have any influence on the study design, data collection, analysis or interpretation of results. The authors do not have any conflict of interest.

For peer review only

References

1 Ahangari A. Prevalence of Chronic Pelvic Pain Among Women: An Updated Review. *Pain Physician* 2014; **17**: E141-E7

2 Daniels JP, Khan KS. Chronic pelvic pain in women. *BMJ* 2010; **341**

3 Howard FM. The role of laparoscopy in chronic pelvic pain: promise and pitfalls. *Obstetrical & gynecological survey* 1993; **48**: 357-87

4 Kaya S, Hermans L, Willems T, Roussel N, Meeus M. Central sensitization in urogynecological chronic pelvic pain: a systematic literature review. *Pain Physician* 2013; **16**: 291-308

5 Eller-Smith OC, Nicol AL, Christianson JA. Potential Mechanisms Underlying Centralized Pain and Emerging Therapeutic Interventions. *Frontiers in Cellular Neuroscience* 2018; **12**

6 Royal College of Obstetricians and Gynaecologists. Therapies Targeting the Nervous System for Chronic Pelvic Pain Relief. 2015. Available from https://http://www.rcog.org.uk/globalassets/documents/guidelines/scientific-impact-papers/sip46.pdf 2015)

7 Royal College of Obstetricians and Gynaecologists. The initial management of chronic pelvic pain. . *Green-top Guideline* 2012

8 Dworkin RH, Peirce-Sandner S, Turk DC, et al. Outcome measures in placebo-controlled trials of osteoarthritis: responsiveness to treatment effects in the REPORT database. *Osteoarthr Cartilage* 2011; **19**: 483-92

9 Wise RG, Tracey I. The role of fMRI in drug discovery. *Journal of Magnetic Resonance Imaging* 2006; **23**: 862-76

10 Horne AW, Critchley HO, Doust A, et al. GaPP: a pilot randomised controlled trial of the efficacy of action of gabapentin for the management of chronic pelvic pain in women: study protocol. *BMJ open* 2012; **2**

11 Lewis SC, Bhattacharya S, Wu O, et al. Gabapentin for the Management of Chronic Pelvic Pain in Women (GaPP1): A Pilot Randomised Controlled Trial. *PloS one* 2016; **11**: e0153037

12 Iannetti GD, Zambreanu L, Wise RG, et al. Pharmacological modulation of pain-related brain activity during normal and central sensitization states in humans. *Proc Natl Acad Sci U S A* 2005; **102**: 18195-200

For peer review only

13 Huang GD, Bull J, Johnston McKee K, et al. Clinical trials recruitment planning: A proposed framework from the Clinical Trials Transformation Initiative. *Contemporary clinical trials* 2018; **66**: 74-9

14 Eldridge SM, Lancaster GA, Campbell MJ, et al. Defining Feasibility and Pilot Studies in Preparation for Randomised Controlled Trials: Development of a Conceptual Framework. *PloS one* 2016; **11**: e0150205

15 Cooper CL, Whitehead A, Pottrill E, Julious SA, Walters SJ. Are pilot trials useful for predicting randomisation and attrition rates in definitive studies: A review of publicly funded trials. *Clinical trials (London, England)* 2018; **15**: 189-96

16 Guo Q, Thabane L, Hall G, McKinnon M, Goeree R, Pullenayegum E. A systematic review of the reporting of sample size calculations and corresponding data components in observational functional magnetic resonance imaging studies. *NeuroImage* 2014; **86**: 172-81

17 Segerdahl AR, Mezue M, Okell TW, Farrar JT, Tracey I. The dorsal posterior insula subserves a fundamental role in human pain. *Nature neuroscience* 2015; **18**: 499-500

18 Lu C, Yang T, Zhao H, et al. Insular Cortex is Critical for the Perception, Modulation, and Chronification of Pain. *Neuroscience bulletin* 2016; **32**: 191-201

19 Brawn J, Morotti M, Zondervan KT, Becker CM, Vincent K. Central changes associated with chronic pelvic pain and endometriosis. *Human reproduction update* 2014; **20**: 737-47

20 Levesque A, Riant T, Ploteau S, Rigaud J, Labat JJ. Clinical Criteria of Central Sensitization in Chronic Pelvic and Perineal Pain (Convergences PP Criteria): Elaboration of a Clinical Evaluation Tool Based on Formal Expert Consensus. *Pain Med* 2018

21 Patrick-Lake B. Patient engagement in clinical trials: The Clinical Trials Transformation Initiative's leadership from theory to practical implementation. *Clinical trials (London, England)* 2018; **15**: 19-22

22 Moayedi M, Salomons TV, Atlas LY. Pain Neuroimaging in Humans: A Primer for Beginners and Non-Imagers. *J Pain* 2018

23 Karos K, Alleva JM, Peters ML. Pain, Please: An Investigation of Sampling Bias in Pain Research. *J Pain* 2018

For peer review only

24 Smith SM, Dworkin RH, Turk DC, et al. The Potential Role of Sensory Testing, Skin Biopsy, and Functional Brain Imaging as Biomarkers in Chronic Pain Clinical Trials: IMMPACT Considerations. *J Pain* 2017; **18**: 757-77

25 Bennett CM, Miller MB. How reliable are the results from functional magnetic resonance imaging? In: Kingstone AMMB, ed. *Year in Cognitive Neuroscience 2010*, 2010; 133-55

26 Cremers HR, Wager TD, Yarkoni T. The relation between statistical power and inference in fMRI. *PloS one* 2017; **12**: e0184923

For peer review only

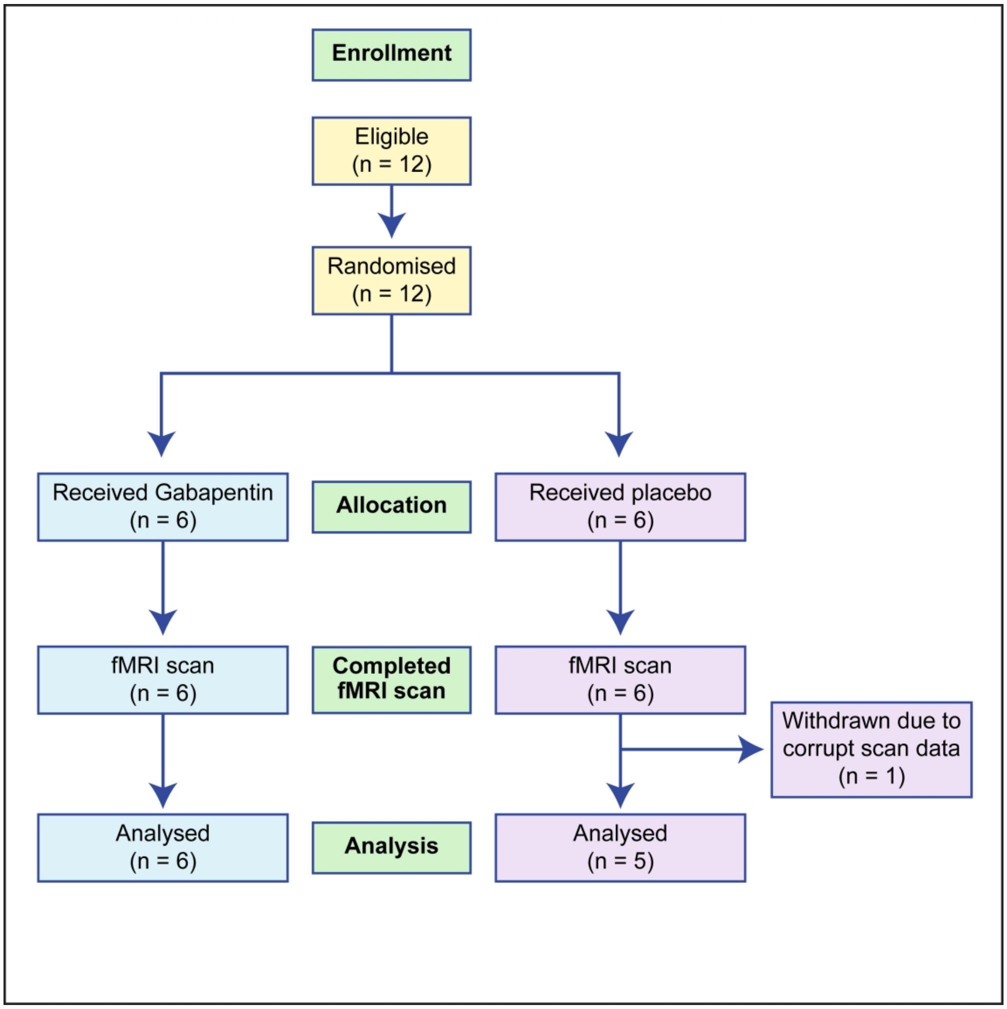

Figure 1. Consort Diagram

149x149mm (600 x 600 DPI)

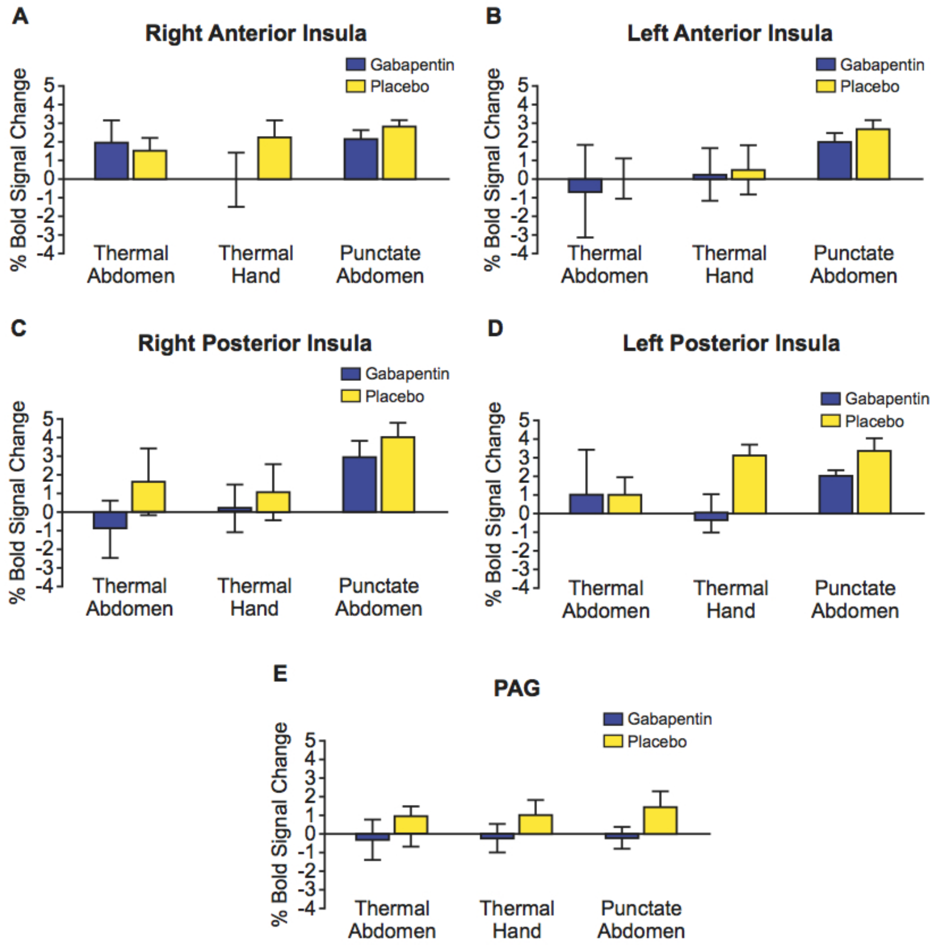

Figure 2. Group differences in BOLD Signal Change in regions of interest
PAG = periaqueductal grey. BOLD = blood oxygen level dependent. Although not statistically significant the analysis showed decreased activation for the gabapentin group in all ROI following all stimuli, except in the RAI and LPI following abdominal noxious heat. The Left PI showed significantly decreased mean activation ($p=0.015$ independent sample, one sided t-test) in response to punctate stimuli on the abdomen and tended towards significance ($p=0.051$) for punctate stimuli of the hand. Our results suggest that gabapentin changes the set point for acute pain modulation in CPP patients. The PI has been shown to have strong connectivity with the somatosensory cortex and is involved in transitioning from painful to non-painful sensations. Significantly decreased pain-related activity in this region in the gabapentin group highlights the potential central efficacy of this drug in women with CPP.

150x150mm (149 x 149 DPI)

BMJ Open

The use of brain functional magnetic resonance imaging to determine the mechanism of action of gabapentin in managing chronic pelvic pain in women: a pilot study

Journal:	*BMJ Open*
Manuscript ID	bmjopen-2018-026152.R1
Article Type:	Research
Date Submitted by the Author:	30-Apr-2019
Complete List of Authors:	Seretny, Marta; University of Edinburgh, Edinburgh Cancer Research UK Centre<br>Murray, Sarah; University of Edinburgh, MRC Centre for Reproductive Health<br>Whitaker, Lucy; University of Edinburgh, MRC Centre for Reproductive Health<br>Murnane, Jonathan; University of Edinburgh, Queens Medical Research Institute and Edinburgh Imaging facility (QMRI)<br>Whalley, Heather; University of Edinburgh, Centre for Clinical Brain Sciences Centre for Genomic and Experimental Medicine<br>Pernet, Cyril; University of Edinburgh, Queens Medical Research Institute and Edinburgh Imaging facility (QMRI)<br>Horne, Andrew
**Primary Subject Heading**:	Obstetrics and gynaecology
Secondary Subject Heading:	Pharmacology and therapeutics
Keywords:	Chronic pelvic pain, Functional Magnetic Resonance Imaging, Pilot Randomised Control Trial (RCT), Gabapentin, Placebo

SCHOLARONE™
Manuscripts

The use of brain functional magnetic resonance imaging to determine the mechanism of action of gabapentin in managing chronic pelvic pain in women: a pilot study

Authors

Marta Seretny MD, MPH, PhD, FRCA University of Edinburgh

Sarah Murray MB ChB, MRCOG University of Edinburgh

Lucy Whitaker MB ChB, MSc, MRCOG, MD University of Edinburgh

Jonathan Murnane BSc, MSc University of Edinburgh

Cyril R Pernet PhD University of Edinburgh

Heather C Whalley University of Edinburgh

*Andrew W Horne MB ChB, PhD, FRCOG University of Edinburgh

*Contact Details Corresponding Author:

Prof Andrew Horne

Email: Andrew.Horne@ed.ac.uk

Address: MRC Centre for Reproductive Health, The University of Edinburgh

Queen's Medical Research Institute

47 Little France Crescent

Edinburgh EH16 4TJ

Phone: +44 131 242 6162

Word Count: 2549

For peer review only

Article Summary

Strengths and Limitations of this study:

- This pilot study includes women with chronic pelvic pain (CPP) randomised to receive placebo or gabapentin for its management.
- All participants in this pilot study underwent a brain fMRI scan.
- This study estimated sample size for a larger fMRI study assessing gabapentin in CPP and also assessed study acceptability.
- The fMRI sample was too small to draw any statistical conclusions, all results are suggestive of trends only.

For peer review only

Key Words:

Chronic Pelvic Pain, Functional Magnetic Resonance Imaging (fMRI), Gabapentin, Placebo, Pilot Randomised Control Trial (RCT), Pilot Study

Abstract

Objective: To inform feasibility and design of a future randomised controlled trial (RCT) using brain functional magnetic resonance imaging (fMRI) to determine the mechanism of action of gabapentin in managing chronic pelvic pain (CPP) in women.

Design: Mechanistic study embedded in pilot RCT.

Setting: University Hospital

Participants: Twelve women (18–50 years) with CPP and no pelvic pathology (follow up completed March 2014).

Intervention: Oral gabapentin (300-2700mg) or matched placebo.

Outcome measures: After 12 weeks of treatment, participants underwent functional MRI (fMRI) of the brain (Verio Siemens 3T MRI) during which noxious heat and punctate stimuli were delivered to the pelvis and arm. Outcome measures included pain (visual analogue scale), Blood Oxygen Level Dependent (BOLD) signal change and a semi structured acceptability questionnaire at study completion prior to unblinding.

Results: Full datasets were obtained for 11 participants. Following noxious heat to the abdomen the gabapentin group (GG) had lower pain scores (Mean: 3.8 [SD 2.2]) than the placebo group (PG) (Mean: 5.8 [SD 0.9]). This was also the case for noxious heat to the arm with the GG having lower pain scores (Mean: 2.6 [SD 2.5]) then the PG (Mean: 6.2 [SD 1.1]). Seven out of 12 participants completed the acceptability questionnaire. 71% (five out of seven) described their participation in the fMRI study as positive; the remaining two rated it as a negative experience.

For peer review only

Conclusions: Incorporating brain fMRI in a future RCT to determine the mechanism of action of gabapentin in managing CPP in women was feasible and acceptable to most women.

Trial Registration: ISRCTN70960777.

For peer review only

Introduction

Chronic pelvic pain (CPP) affects >3.3 billion women worldwide and costs an estimated >$225 million/year (£150 million) in socioeconomic costs in the UK alone[1]. Underlying pathology, such as endometriosis or adhesions, can be identified in around 65% of women with CPP. In the remaining 35% of women, pain symptoms have no clear aetiology[2 3]. It is increasingly recognised that women with CPP of unknown aetiology likely represent a subgroup of patients who have undergone central sensitisation (CS), an increased responsiveness of central nociceptive neurons to their normal or sub-threshold afferent input[4]. The treatment for women with CPP of unknown aetiology is challenging[5].

Currently there are no evidence-based analgesics for CPP[6] but gabapentin (a GABA analogue, eliciting its analgesic effects via the spinal N-type calcium channel[7]) is increasingly prescribed due to its known effectiveness in other neuropathic pain states[6]. The true efficacy, mechanism of action and side effect profile of gabapentin in women with CPP remains unknown. However, in general investigation of analgesic drug efficacy in clinical trials is not straightforward. Due to the subjective nature of pain and the influence of an active physiological placebo response, trials investigating analgesics, even when appropriately powered and carefully executed, often produce small effect sizes that are difficult to interpret clinically [8]. Nevertheless, the increase in the use of adjuvant research tools, such as brain functional magnetic resonance imaging (fMRI), has been shown to strengthen and mechanistically explain findings[9].

For peer review only

We therefore undertook a pilot exploratory study to determine the feasibility of investigating the mechanism of action of gabapentin in managing women with CPP using brain fMRI. The objectives of the study were to:

1) To determine whether it is possible to recruit women with CPP, who had agreed to participate in a related pilot clinical trial and received gabapentin or placebo, to partake in an embedded mechanistic study and undergo a brain fMRI scan.

2) To analyze the fMRI brain data and use it for sample size calculation for a future large fMRI brain study investigating the mechanism of action of gabapentin in managing CPP in women.

3) To assess if the fMRI and associated noxious stimuli were acceptable to participants.

For peer review only

Materials and Methods

Study design

This was a pilot exploratory mechanistic study. The flow of patients in the study is detailed in a CONSORT diagram (see figure 1).

Setting

Women with CPP in NHS Lothian (UK), already recruited as part of a pilot RCT conducted in NHS Lothian and NHS Grampian (UK) to investigate the efficacy of gabapentin versus placebo in CPP management[10 11], were approached and invited to participate in this embedded fMRI substudy. The pilot RCT aimed to enrol 60 participants over a nine-month period. The brain fMRI scans were carried out on a Siemens Verio 3T MRI Scanner located at the Edinburgh Imaging Facility, Queens Medical Research Institute (previously known as the Edinburgh Clinical Research Imaging Centre), at the University of Edinburgh (UK). A 12 channel receiver head coil was used. Other study measures were tested in NHS Lothian clinical research facilities or, where appropriate, via the telephone.

For peer review only

Inclusion criteria

- Women aged between 18 and 50 years
- Chronic pelvic pain (non-cyclical pain with or without dysmenorrhoea or dyspareunia) assessed clinically to be located within the true pelvis or between and below anterior iliac crests[12]
- Associated functional disability

- No obvious pelvic pathology at laparoscopy (<36 months and >2 weeks ago)
- Normal pelvic ultrasound (<36 months)
- Using effective contraception e.g. barrier methods, combined oral contraceptive pill, etc.
- Written informed consent

Exclusion criteria

- Known pelvic pathology (for example, endometriosis, cyst, fibroid>3cm)
- Past history of having taken gabapentin or pregabalin
- Due to undergo surgery in the next six months
- History of significant renal impairment
- Allergic to gabapentin or excipients
- Breast feeding
- Pregnancy or planning pregnancy in the next six months
- Metal implant/pacemaker/claustrophobia (fMRI subgroup).

Intervention

Patients were randomised to receive gabapentin at an increasing dose (300-2700mg) or identical placebo tablet. Gabapentin was started at 300mg daily and titrated up weekly, in 300mg increments or until the patient perceived a 50% reduction in pain, or had side effects, to a maximum of 2700mg. Full details are published in the related clinical trial protocol and results[10 11].

For peer review only

Outcome Measures

Women underwent an fMRI scan of the brain after a minimum of twelve weeks of treatment, to ensurine that all participants had reached their steady state dosing regimen at the time of scanning. The following sequences were collected: structural MPRAGE sequence, resting state BOLD sequence where participants were asked to fixate on a cross, BOLD sequences during which punctate and noxious heat stimuli were applied to the arm and lower abdomen respectively. Full details of MRI acquisition parameters are shown in table 1. The order of the presented stimuli was randomised (some participants had thermal stimulation applied to the arm then abdomen, whilst others had it applied in the reverse order). Thermal stimulus was individually thresholded outside the scanner for each participant. For the arm, a *MEDOC Pathway* thermode was placed on the inner arm, three fingerbreadths proximal to the wrist joint. Temperature was increased from 30C until the patient reported a pain score of five out of ten. The same was repeated for the lower abdomen (three finger breadths above the pubic bone) until the patient reported a pain score of five out of ten. These temperatures were used in the scanner in a block experimental design; temperature at neutral for 10seconds and then increased to the participant's pre-established noxious heat threshold and held for 15seconds and then returned to neutral). The duration of the thermal blocks was ten minutes on the arm and ten minutes on the lower abdomen. Pain scores were recorded in real time during the application of the thermal stimulus using a visual analogue scale presented to participants on a screen inside the scanner. Each participant used an automated pointer to rate her pain. Standardised punctate stimuli were applied to the lower abdomen, three fingerbreadths above the pubic bone using a 300g *Touch*

For peer review only

Test^{TM} von Frey filament (6.65mm) in an event related experimental design. The full duration of the punctate experiment was ten minutes.

**Weighting**	**T1 weighted image**	**Functional MRI (T2*)**
Pulse sequence	Inversion Recovery	Fast Gradient echo
Imaging type	MPRAGE	Echo Plannar Imaging
Flip angle	9	90
Echo time (TE)	2.98 ms	30 ms
Repetition time (TR)	2.3 sec	2.5 sec
Number of volumes	n/a	Thermal runs: 245<br>Punctate run: 260<br>Resting state: 200
Number of discarded volumes	n/a	0
Phase encoding	Anterior / Posterior	Anterior / Posterior
Parallel imaging	GRAPPA *2	GRAPPA *2
Slice order and timing	Interleaved	Interleaved
Slice position procedure	ACPC	ACPC
Brain Coverage	Full brain	Full brain
Field of View	256	192
Matrix size	256 * 256	64*64
Slice Thickness	1 mm	3
Prospective Motion correction	None	On

Table 1. MRI acquisition parameters. Details provided follow recommendations of the COBIDAS report (http://biorxiv.org/content/early/2016/07/10/054262)

For peer review only

To assess the acceptability of the fMRI substudy, participating women were invited to complete a semi-structured questionnaire administered by telephone at the conclusion of the study protocol prior to unblinding. The questions were designed to assess the participant's feelings and experiences related to the fMRI scan (table 2). The responses were recorded on a structured coding sheet. Respondents were asked to answer each question using a five-point positive (one) to negative (five) scale, with the option of adding free comments.

**Regarding the fMRI substudy**	**Positive**				**Negative**
	1	2	3	4	5
A. … was the choice to opt-in or out a positive or negative one? Did you feel pressured?					
B. … did you feel you were fully informed as to why you were having it?					
C. … was the fMRI a comfortable experience?					
D. … how did you find having the blood sample taken?					
E. … experiences and thoughts on the pain threshold exam					

Table 2. Semi Structured Study Acceptability Questionnaire. Women were asked these questions during a phone interview-taking place prior to study unblinding. The researcher recorded free comments elaborating each question.

Statistical Analysis

All statistical analysis was carried out prior to participant and clinician unblinding. FMRI statistical analysis was carried out using *Statistical Parametric Mapping* (SPM12) software[13]. Functional MRI data first underwent a slice timing correction (interpolation based on a fast Fourier transform FFT, 45th slice as reference), followed by motion correction (six degrees affine registration minimizing the mean

For peer review only

square difference, realigned to the first scan of each session then to the mean, fourth degree B-spline interpolation). The T1 weighted image was enhanced using SUSAN[14] from *FMRIB Software Library* (FSL). Images were then coregistred to the mean EPI image (12 degrees affine transformation maximizing the normalized mutual information with no interpolation applied) and then simultaneously segmented/bias corrected to derive normalization parameters to the MNI space[15]. Volume based inter-subject registration was next performed applying the normalization parameters to all EPI images (fourth degree B-spline interpolation, final voxel size [2 2 2]). These images were finally smoothed with a 6mm isotropic Gaussian kernel. For the first level analysis the stimuli of each session were modelled convolving stimulus onsets with a double gamma haemodynamic response function with derivates[16] and durations of zero seconds. Design matrices also included standard six motion parameters and filters for low frequency (high pass filter 128 seconds) and high frequency noise (first order auto-regressive plus Gaussian whitening). For the thermal stimuli, presentation scales were also modelled for design completeness but not investigated. Regions of interest (ROIs) were defined using NeuroSynth[17] with the keywords chronic pain (http://neurosynth.org/analyses/terms/chronic%20pain/). The mean beta values among voxels in 6mm spheres centred on the prefrontal thalamus ([+/-10 -15 4]), the insulae (Iq2 [+/-37 -20 14]), the peri-acqueducal gray (PAG - [0 23 -17]) and whole brain stem ([0 -39 54]). These were obtained for each subject and group comparison was performed for each ROI (percentile bootstrap on median difference, p=0.05 Bonferroni corrected – Wilcox, 2015). Power calculations to calculate the sample size needed for 80% power at a significance level of p=0.05, were conducted

For peer review only - http://bmjopen.bmj.com/site/about/guidelines.xhtml

using the *GPower 3.1* program[18]. The entire dataset was prepared following the Brain Imaging Data Structure (BIDS)[19] and is available on the University of Edinburgh DataShare repository (Horne et al., 2018 https://doi.org/10.7488/ds/2411). Statistical maps are available on NeuroVault[20] (https://neurovault.org/collections/5029/).

Qualitative Analysis

Questionnaire data was assessed thematically in order to identify participant's feelings and experiences related to the fMRI scan and associated noxious heat thresholding. Free comments were reviewed to gain greater understanding on themes important to individual women in the study.

Ethical Approval

The Scotland A Research Ethics committee gave approval for this study (REC 12/SS/0005) on November 13th 2012. All participants gave written informed consent prior to taking part in this study. Consent was also reconfirmed verbally before every fMRI scan.

Patient and Public Involvement

The patient co-founder of the Pelvic Pain Support Network helped with the design of this study and also assisted with gathering feedback regarding the proposed study within the charity. A short description of the proposed pilot study, planned resulting RCT and a series of related questions were distributed amongst members.

For peer review only

Encouragingly, from a survey of 85 women involved in the charity, 69% responds said that they would consider taking part in this pilot.

Results

Recruitment

137 women fulfilled the eligibility criteria for the main pilot clinical trial, and 47 women were randomised to treatment[11]. Of the 47 women who were randomised, Women taking part in the RCT at the Edinburgh University Hospital were offered participation in the fMRI sub-study. 12 consecutive participants were approached to take part in the fMRI sub-study. All of these women consented to undergo a brain fMRI scan, completing the full scanning protocol. Their baseline demographics are shown in table 3. One subject (on placebo) was excluded from analysis following discovery of corrupted scan data at the analysis stage of the study. Progression of pain ratings throughout the study, maximum dose of gabapentin achieved, time to reach maximum dose and reported side effects are summarised in table 4.

For peer review only

Characteristic	Gabapentin (% of 6 or 95% CI)	Placebo (% of 6 or 95% CI)
Age (years)	31.7 (20.3 – 43.0)	30.3 (23.1 – 37.5)
Pain Score (0-10)	6.6 (5.8 – 7.5)	5.5 (3.3– 7.7)
BMI (kg/m²)	25.05 (21.0 – 29.1)	26.05 (21.2– 30.9)
HADS score (max score 21)	9.7 (6.6 – 12.7)	8 (3.4 -12.5)
Parity:<br>Nulliparous<br>Parous	 4 (66.7%)<br>2 (33.3%)	 4(66.7%))<br>2(33.3%))
Higher Education Received:<br>Highschool<br>College/University	 0(0%)<br>6(100%)	 1(16.7%)<br>5(83.3%)

Table 3. Demographics of women who underwent fMRI brain. HADS= hospital anxiety and depression score.

ID	Group	Baseline Pain	3mo Pain	6mo Pain	Max Dose (mg)	Time to Max Dose	Side effects noted
0104	Active	7	4	3	1800	6	Dry mouth, itchy skin
0107	Active	7	6	4	2100	6	Tired
0113	Placebo	6	6	4	600	8	Tired, dizzy, nightmares
0114	Placebo	6	4	6	2400	8	Dizzy, nausea
0116	Placebo	7	4	5	2400	12	Dizzy, nausea
0119	Placebo	5	4	6	300	*	Dizzy, nausea
0120	Placebo	5	2	3	600	1	Tired, dizzy
0122	Placebo	2	1	2	2100	15	None
0123	Active	8	7	6	2400	12	Restlessness
0124	Placebo	8	7	5	1800	12	Low mood, nausea
0125	Active	6	3	2	1800	8	Dizzy, nausea
0129	Active	6	4	3	1800	7	None

Table 4. Showing pain score (0-10 scale) changes during treatment, maximum treatment dose as well as reported side effects. ID= anonymised study ID number. Active= gabapentin recipients. Time to max dose in weeks. *=missing data

For peer review only

fMRI Analysis

fMRI brain scan data for six subjects receiving gabapentin and five receiving placebo was analysed. Average pain ratings during the fMRI scan following noxious stimuli of the abdomen were lower for the gabapentin group (Mean: 3.8 [SD 2.2]) than the placebo group (Mean: 5.8 [SD 0.9]). This was also the case for noxious heat applied to the arm where the gabapentin group had lower average pain scores (Mean: 2.6 [SD 2.5]) than the placebo group (Mean: 6.2 [SD 1.1]). Figure 2 available at https://drive.google.com/open?id=1Iroa8uxXLQ-w6-kBPbVtqPagLjTjtuPu shows unthresholded activation maps for each of the presented stimuli as well as ROIs and the statistical comparison between these. To summarise, the ROI analysis showed BOLD signal changes in the left PI following punctate stimuli, was significantly different between those receiving placebo as compared to those receiving gabapentin (figure 2), this was also the case for activation in the PAG following heat application to the lower abdomen. All other ROI comparisons showed no statistically significant differences (tables 5,6,7). As expected, due to the exploratory sample size, group level whole brain analysis did not yield any statistically significant between group differences.

For peer review only

Region	Gabapentin	Placebo	Difference	P value
Left Insula	-0.14 [-1.71 0.44]	-0.23 [-1.01 0.14]	[-0.98 0.82]	0.8820
Right Insula	-0.38 [-0.52 0.01]	-0.19 [-0.51 0.13]	[-0.54 0.32]	0.5160
Left Thalamus	0.97 [-0.28 2.44]	1.97 [-1.12 3.31]	[-2.53 1.4]	0.3620
Right Thalamus	-0.11 [-0.83 0.03]	-0.08 [-0.37 0.66]	[-0.84 0.23]	0.7360
PAG	-0.35 [-0.7 -0.004]	0.0004 [-0.07 0.09]	[-0.61 -0.05]	**0.004**
Brain Stem	-0.3579 [-1.25 0.21]	-0.52 [-3.71 0.61]	[-1 2.5]	0.7900

Table 5. ROI median beta values with 95% highest density intervals following thermal stimulation to the abdomen. Please note difference = percentile bootstrap median difference and associated p value of this.

Region	Gabapentin	Placebo	Difference	P value
Left Insula	0.01 [-1 0.52]	0.19 [-0.07 0.4]	[-0.95 0.25]	0.454
Right Insula	-0.13 [-0.33 0.1]	0.14 [-0.42 0.39]	[-0.55 0.22]	0.254
Left Thalamus	0.53 [-0.44 0.88]	1.68 [-0.2 2.34]	[-2.01 0.1]	0.07
Right Thalamus	-0.09 [-1.03 0.4]	0.13 [-0.01 0.3]	[-0.91 0.21]	0.388
PAG	-0.11 [-0.42 0.1]	-0.05 [-0.5 0.29]	[-0.42 0.36]	0.84
Brain Stem	0.5 [-0.42 1.16]	1.8 [-0.52 2.72]	[-2.23 0.28]	0.096

Table 6. ROI median beta values with 95% highest density intervals following thermal stimulation to the hand. Please note difference = percentile bootstrap median difference and associated p value of this.

For peer review only

Region	Gabapentin	Placebo	Difference	P value
Left Insula	0.14 [-0.35 0.54]	-0.33 [-1 -0.1]	[0.1 1.07]	**0.042**
Right Insula	-0.24 [-0.71 0.26]	0.25 [-0.65 -0.018]	[-0.3 0.6]	0.892
Left Thalamus	-0.95 [-2.5 -0.14]	-0.34 [-1.28 1.99]	[-3.45 0.2]	0.114
Right Thalamus	-0.11 [-1.99 0.72]	-0.09 [-0.14 -0.06]	[-1.1 0.7]	0.948
PAG	-0.05 [-0.45 0.16]	-0.21 [-1.16 0.39]	[-0.4 0.99]	0.654
Brain Stem	-0.54 [-1.59 1.01]	0.12 [-4.63 1.62]	[-2.1 2.49]	0.65

Table 7. ROI median beta values with 95% highest density intervals following punctate stimulation to the lower abdomen. Please note difference = percentile bootstrap median difference and associated p value of this.

A sample size calculation from these results, suggest that for a mechanistic sub study embedded in a larger double blind RCT investigating the efficacy of gabapentin versus placebo in CPP, a minimum of 7 women per group (a total of 14 brain fMRI scans) are required for 80% power and p=0.05 significance level, sampling ratio 1:1, with two independent samples and two-sided testing. This calculation was based on differences in BOLD signal activation in the left insula (punctate) and PAG (abdomen thermal stimuli) or if averaging across all ROI and all conditions.

FMRI Substudy Acceptability

All twelve women were approached for the telephone interview. Seven were contactable and gave consent to the telephone administered semi- structured acceptability questionnaire. Five women rated the fMRI as a positive experience, scoring it as 1 or 2 on a 1 - 5 point, positive to negative scale. Two participants

For peer review only

scored their experience as ‘four’ on the scale. One of these commented that the trial substudy process was not explained clearly enough. This participant was also disappointed by lack of feedback after the scan. The other participant rating the experience negatively felt that an in-person explanation rather than the information leaflet would have been clearer. All participants felt the option of opting in or out of the mechanistic fMRI substudy was positive. The majority of women found the experience of the actual scan positive or neutral, with similar ratings given to the quality of information given before the scan, as well as their experience of the noxious pain thresholding. One participant noted that prior to the study she had anxiety related to MRI scans and that study participation actually helped her overcome her fear of MRI. Only one participant felt that the information given before the scan, the actual scan experience, as well as the noxious thresholding was a negative experience for her (a score of four on the five-point scale).

Discussion

Recruitment

Our study met our objectives in relation to establishing feasibility of recruitment. All of the participants approached for brain fMRI consented to this part of the study and completed the full hour-long protocol in the scanner. Failure to recruit to trials is known to be a major limitation in clinical research, prompting new recommendations aimed at improving approaches to recruitment[21]. Assessment of study feasibility is a part of this improvement process. Pilot studies are considered a subtype of formal feasibility assessments[22]. Further, pilot studies are deemed valuable in ascertaining unknown factors such as recruitment to novel outcome

For peer review only

measures, such as in our case brain fMRI in CPP patients[23]. We feel our successful recruitment to this study supports future fMRI research investigating the mechanism of action of gabapentin for the management of women with CPP.

fMRI Analysis

The key outcome of our fMRI data analysis was calculation of sample size for a planned future brain fMRI study to investigate the mechanism of action of gabapentin in the management of women with CPP. Sample size calculations for fMRI studies have become a standard part of good MRI research methodology[24]. We also explored BOLD signal changes in response to punctate and noxious heat stimuli, in regions of the brain known to be involved in pain processing. Our findings suggest between group differences in response to punctate and abdominal heat stimuli in the insula and PAG. These trends fit with what is known about the fundamental role of the insula and the PAG in central sensitisation and pain processing[25 26]. Specifically, in CPP both the PAG and insula are known to have altered structure and connectivity[27 28 29]. Moreover, central sensitisation is postulated to be an important mechanism in the maintenance of CPP[30 31]. Neuromodulators such as gabapentin have been shown in neuroimaging studies to modify central sensitisation pain states[32]. Further work in a larger mechanistic fMRI study is needed to confirm and elucidate these findings.

Study Acceptability

Patient engagement in clinical trials is known to improve study recruitment and retention[33]. Specific to pain research and analgesic RCTs, many issues limit patient

For peer review only - http://bmjopen.bmj.com/site/about/guidelines.xhtml

participation in pain trials utilising fMRI, not least the actual experience of chronic pain[34]. Decreased participation of subsets of pain patients in pain medication trials can introduce potential biases into study findings [35]. Understanding patient experiences, which in our case helped highlight the need for clearer communication around study logistics, will help utilise fMRI in future mechanistic probing of gabapentin efficacy in CPP. This is particularly valuable, as the utility of fMRI in chronic pain management research is deemed to have growing importance[36].

Study Strengths and Limitations

The major limitation of our fMRI data analysis lies in our small sample size. Approaches to brain fMRI analysis are varied and maybe prone to false positive or non-reproducible results[37]. This is particularly true when small datasets are used [38]. It is therefore possible that a different approach to the analysis of our data may have yielded different results. We therefore interpret these findings as interesting trends, in need of further validation, and we have made our fMRI dataset available to interested researchers who may wish to re analyse our data or combine it with another relevant dataset. Another limitation of our study is lack of data regarding the duration of CPP in study participants, we have corrected this in the ongoing GaPP2 study. The strengths of this study relate to all the study aims being fulfilled and clearly reported. This study is one of the first to utilise fMRI in the assessment of women with CPP receiving gabapentin or placebo.

For peer review only

Conclusion

We have demonstrated that it is possible to recruit women with CPP participating in a pilot RCT of gabapentin versus placebo, to take part in a mechanistic substudy involving a brain fMRI scan. Most participants in our pilot found the MRI scan, including administration of noxious stimuli acceptable. BOLD signal changes identified in our analysis are likely useful guides for further studies aimed at understanding the mechanism of action of gabapentin in CPP. Sample size calculations resulting from this work have been used to inform the design of a mechanistic fMRI substudy in a larger RCT investigating gabapentin versus placebo for the management of CPP in women.

For peer review only

Figure Legends

Figure 1. CONSORT diagram

Figure 2. Activation maps (unthresholded) for each stimulus presented, alongside ROI group comparisons. Available at **https://drive.google.com/file/d/1Iroa8uxXLQ-w6-kBPbVtqPagLjTjtuPu/view**

For peer review only

Conflict of Interest

We have read and understood the BMJ Open policy on declaration of interests and declare that we have no competing interests

Author Statement

MS, AH, CP where involved in the design and set up of the study. MS, LW, SM, JM and CP were involved in scan data collection. CP analysed the fMRI data. CP and HW performed the sample size calculation. All authors contributed to the writing of this manuscript.

Data Statement

All data from this study is currently held securely at the University of Edinburgh. Anonymised fMRI data is available to interested researchers and can be accessed at: https://datashare.is.ed.ac.uk/handle/10283/3153

Acknowledgments

We thank Katy Vincent (DPhil, BSc, MBBS, MRCOG) who was instrumental in guiding the set up and data analysis for this study. Thank you to Scott Semple (PhD) and Neil Roberts (PhD), MRI physicists for their assistance in setting up additional data acquisition equipment and monitoring in the MRI scanner. We would also like to thank Ms Jennifer Brawn (MSc, DPhil) for organising scan sequence transfer and set up between Oxford and Edinburgh. We thank all the radiographers at the Clinical Research Imaging Centre (CRIC) Edinburgh, for their contribution to data collection. We also thank Mrs Ann Doust and Mrs Helen Dewart for organising patient

For peer review only

recruitment and follow up. Additional thanks are owed to Ann Doust for managing all study data and answering endless queries related to writing this manuscript. We would like to thank Judy Birch JB, the patient liaison and representative of the Pelvic Pain Support Network for her advice and input into this study. Finally, and perhaps most importantly we would like to thank all the women who took part in this study.

Funding

This work was supported by a grant from the Chief Scientist's Office Scotland (CZH/4/688) and an MRC Centre Grant Mr/N022556/1. MS was funded by the Wellcome Trust via the Scottish Translational Medicine and Therapeutics Initiative (STMTI). JM held a PhD studentship funded by the Mentholatum company. The funders did not have any influence on the study design, data collection, analysis or interpretation of results. The authors do not have any conflict of interest.

For peer review only

References

1. Ahangari A. Prevalence of Chronic Pelvic Pain Among Women: An Updated Review. *Pain Physician* 2014;17(2):E141-E47.

2. Daniels JP, Khan KS. Chronic pelvic pain in women. *BMJ* 2010;341 doi: 10.1136/bmj.c4834

3. Howard FM. The role of laparoscopy in chronic pelvic pain: promise and pitfalls. *Obstetrical & gynecological survey* 1993;48(6):357-87. [published Online First: 1993/06/01]

4. Kaya S, Hermans L, Willems T, et al. Central sensitization in urogynecological chronic pelvic pain: a systematic literature review. *Pain Physician* 2013;16(4):291-308. [published Online First: 2013/07/24]

5. Eller-Smith OC, Nicol AL, Christianson JA. Potential Mechanisms Underlying Centralized Pain and Emerging Therapeutic Interventions. *Frontiers in Cellular Neuroscience* 2018;12(35) doi: 10.3389/fncel.2018.00035

6. Royal College of Obstetricians and Gynaecologists. Therapies Targeting the Nervous System for Chronic Pelvic Pain Relief. 2015 [Available from: https://www.rcog.org.uk/globalassets/documents/guidelines/scientific-impact-papers/sip46.pdf2015.

7. Cheng JK, Chiou LC. Mechanisms of the antinociceptive action of gabapentin. *Journal of pharmacological sciences* 2006;100(5):471-86. [published Online First: 2006/02/14]

8. Dworkin RH, Peirce-Sandner S, Turk DC, et al. Outcome measures in placebo-controlled trials of osteoarthritis: responsiveness to treatment effects in the REPORT database. *Osteoarthr Cartilage* 2011;19(5):483-92.

9. Wise RG, Tracey I. The role of fMRI in drug discovery. *Journal of Magnetic Resonance Imaging* 2006;23(6):862-76. doi: 10.1002/jmri.20584

10. Horne AW, Critchley HO, Doust A, et al. GaPP: a pilot randomised controlled trial of the efficacy of action of gabapentin for the management of chronic pelvic pain in women: study protocol. *BMJ open* 2012;2(3) doi: 10.1136/bmjopen-2012-001297 [published Online First: 2012/06/12]

11. Lewis SC, Bhattacharya S, Wu O, et al. Gabapentin for the Management of Chronic Pelvic Pain in Women (GaPP1): A Pilot Randomised Controlled Trial. *PloS one* 2016;11(4):e0153037. doi: 10.1371/journal.pone.0153037

For peer review only

12. RCOG Green-top Guideline. The Initial Managment of Chronic Pelvic Pain, 2012:1-16.

13. Flandin G, Friston K. Statistical parametric mapping (SPM). *Scholarpedia,* 2008;3(4):6332. doi: https://doi.org/10.4249/scholarpedia.6232

14. Smith SM, Brady JM. SUSAN - a new approach to low level image processing. . *International Journal of Computer Vision* 1997;23(1):45-78. doi: https://doi.org/10.1023/A:1007963824710

15. Ashburner J, Friston KJ. Unified segmentation. *NeuroImage* 2005;26(3):839-51. doi: 10.1016/j.neuroimage.2005.02.018 [published Online First: 2005/06/16]

16. Friston KJ, Holmes AP, Worsley KJ, et al. Statistical parametric maps in functional imaging: A general linear approach. *Human Brain Mapping* 1994;2(4):189-210. doi: 10.1002/hbm.460020402

17. Yarkoni T, Poldrack RA, Nichols TE, et al. Large-scale automated synthesis of human functional neuroimaging data. *Nat Methods* 2011;8(8):665-70. doi: 10.1038/nmeth.1635 [published Online First: 2011/06/28]

18. Faul F, Erdfelder E, Lang A-G, et al. G*Power 3: A flexible statistical power analysis program for the social, behavioral, and biomedical sciences. *Behavior Research Methods* 2007;39(2):175-91. doi: 10.3758/bf03193146

19. Gorgolewski KJ, Auer T, Calhoun VD, et al. The brain imaging data structure, a format for organizing and describing outputs of neuroimaging experiments. *Scientific data* 2016;3:160044. doi: 10.1038/sdata.2016.44 [published Online First: 2016/06/22]

20. Gorgolewski KJ, Varoquaux G, Rivera G, et al. NeuroVault.org: a web-based repository for collecting and sharing unthresholded statistical maps of the human brain. *Frontiers in neuroinformatics* 2015;9:8. doi: 10.3389/fninf.2015.00008 [published Online First: 2015/04/29]

21. Huang GD, Bull J, Johnston McKee K, et al. Clinical trials recruitment planning: A proposed framework from the Clinical Trials Transformation Initiative. *Contemporary clinical trials* 2018;66:74-79. doi: 10.1016/j.cct.2018.01.003

22. Eldridge SM, Lancaster GA, Campbell MJ, et al. Defining Feasibility and Pilot Studies in Preparation for Randomised Controlled Trials: Development of a Conceptual Framework. *PloS one* 2016;11(3):e0150205. doi: 10.1371/journal.pone.0150205

For peer review only

23. Cooper CL, Whitehead A, Pottrill E, et al. Are pilot trials useful for predicting randomisation and attrition rates in definitive studies: A review of publicly funded trials. *Clinical trials (London, England)* 2018;15(2):189-96. doi: 10.1177/1740774517752113

24. Guo Q, Thabane L, Hall G, et al. A systematic review of the reporting of sample size calculations and corresponding data components in observational functional magnetic resonance imaging studies. *NeuroImage* 2014;86:172-81. doi: 10.1016/j.neuroimage.2013.08.012

25. Segerdahl AR, Mezue M, Okell TW, et al. The dorsal posterior insula subserves a fundamental role in human pain. *Nature neuroscience* 2015;18(4):499-500. doi: 10.1038/nn.3969 [published Online First: 2015/03/10]

26. Lu C, Yang T, Zhao H, et al. Insular Cortex is Critical for the Perception, Modulation, and Chronification of Pain. *Neuroscience bulletin* 2016;32(2):191-201. doi: 10.1007/s12264-016-0016-y

27. Brawn J, Morotti M, Zondervan KT, et al. Central changes associated with chronic pelvic pain and endometriosis. *Human reproduction update* 2014;20(5):737-47. doi: 10.1093/humupd/dmu025

28. As-Sanie S, Harris RE, Napadow V, et al. Changes in regional gray matter volume in women with chronic pelvic pain: A voxel-based morphometry study. *Pain* 2012;153(5):1006-14. doi: Doi 10.1016/J.Pain.2012.01.032

29. As-Sanie S, Kim J, Schmidt-Wilcke T, et al. Functional Connectivity is Associated With Altered Brain Chemistry in Women With Endometriosis-Associated Chronic Pelvic Pain. *J Pain* 2016;17(1):1-13. doi: 10.1016/j.jpain.2015.09.008 [published Online First: 2015/10/13]

30. Grundstrom H, Gerdle B, Alehagen S, et al. Reduced pain thresholds and signs of sensitization in women with persistent pelvic pain and suspected endometriosis. *Acta obstetricia et gynecologica Scandinavica* 2019;98(3):327-36. doi: 10.1111/aogs.13508 [published Online First: 2018/11/26]

31. Levesque A, Riant T, Ploteau S, et al. Clinical Criteria of Central Sensitization in Chronic Pelvic and Perineal Pain (Convergences PP Criteria): Elaboration of a Clinical Evaluation Tool Based on Formal Expert Consensus. *Pain Med* 2018 doi: 10.1093/pm/pny030 [published Online First: 2018/03/10]

32. Iannetti GD, Zambreanu L, Wise RG, et al. Pharmacological modulation of pain-related brain activity during normal and central sensitization states in humans. *Proc Natl Acad Sci U S A* 2005;102(50):18195-200. doi: 10.1073/pnas.0506624102

For peer review only

33. Patrick-Lake B. Patient engagement in clinical trials: The Clinical Trials Transformation Initiative's leadership from theory to practical implementation. *Clinical trials (London, England)* 2018;15(1_suppl):19-22. doi: 10.1177/1740774518755055

34. Moayedi M, Salomons TV, Atlas LY. Pain Neuroimaging in Humans: A Primer for Beginners and Non-Imagers. *J Pain* 2018 doi: 10.1016/j.jpain.2018.03.011

35. Karos K, Alleva JM, Peters ML. Pain, Please: An Investigation of Sampling Bias in Pain Research. *J Pain* 2018 doi: 10.1016/j.jpain.2018.02.011 [published Online First: 2018/03/09]

36. Smith SM, Dworkin RH, Turk DC, et al. The Potential Role of Sensory Testing, Skin Biopsy, and Functional Brain Imaging as Biomarkers in Chronic Pain Clinical Trials: IMMPACT Considerations. *J Pain* 2017;18(7):757-77. doi: 10.1016/j.jpain.2017.02.429

37. Bennett CM, Miller MB. How reliable are the results from functional magnetic resonance imaging? In: Kingstone AMMB, ed. Year in Cognitive Neuroscience2010:133-55.

38. Cremers HR, Wager TD, Yarkoni T. The relation between statistical power and inference in fMRI. *PloS one* 2017;12(11):e0184923. doi: 10.1371/journal.pone.0184923

For peer review only

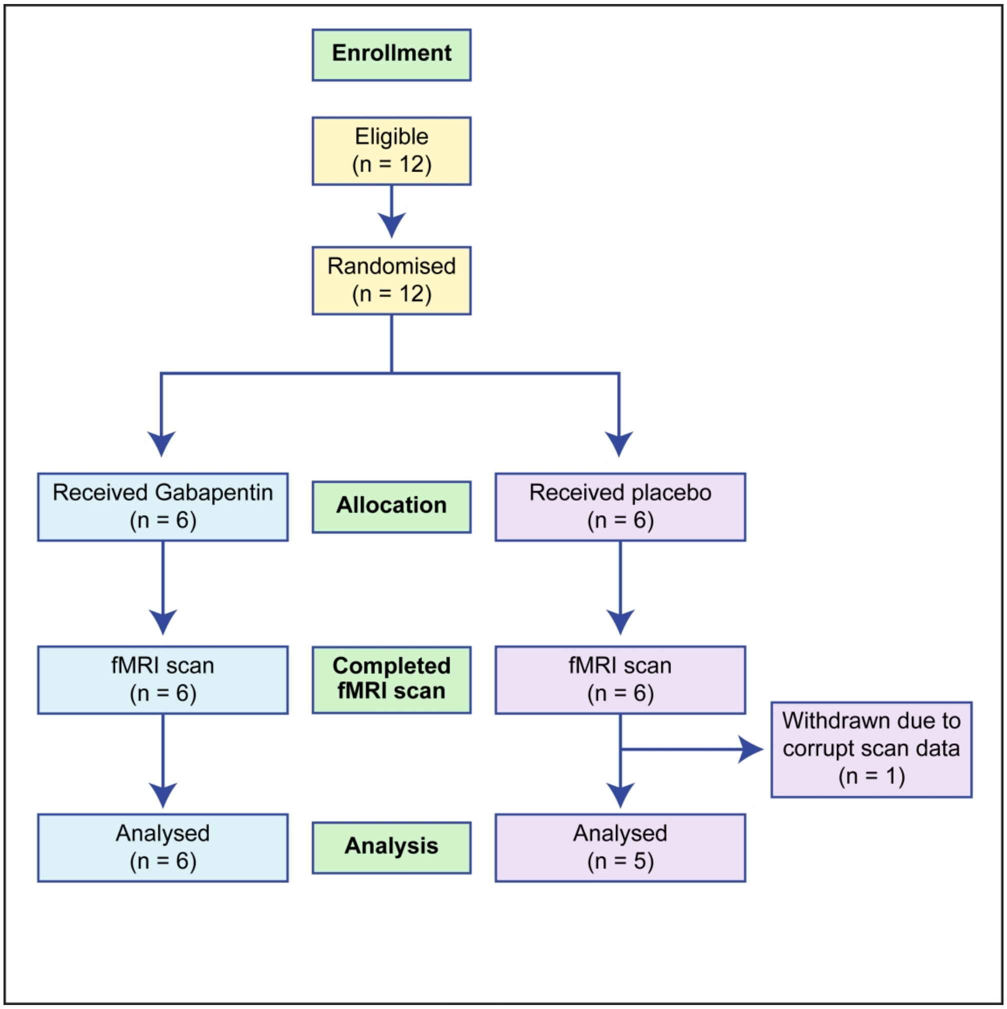

Figure 1. Consort Diagram

149x149mm (600 x 600 DPI)

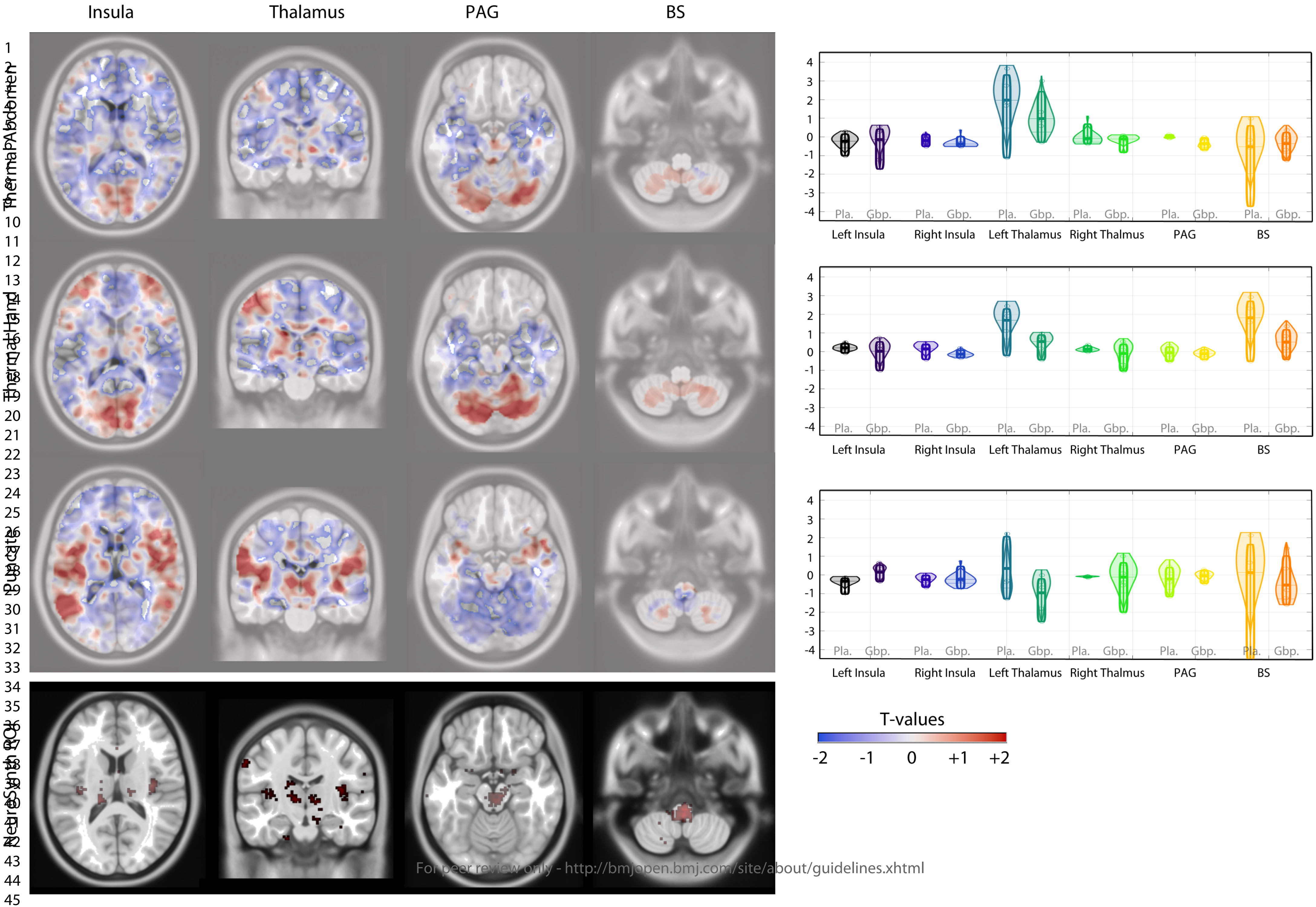

For peer review only - http://bmjopen.bmj.com/site/about/guidelines.xhtml

BMJ Open

The use of brain functional magnetic resonance imaging to determine the mechanism of action of gabapentin in managing chronic pelvic pain in women: a pilot study

Journal:	*BMJ Open*
Manuscript ID	bmjopen-2018-026152.R2
Article Type:	Research
Date Submitted by the Author:	15-May-2019
Complete List of Authors:	Seretny, Marta; University of Edinburgh, Edinburgh Cancer Research UK Centre<br>Murray, Sarah; University of Edinburgh, MRC Centre for Reproductive Health<br>Whitaker, Lucy; University of Edinburgh, MRC Centre for Reproductive Health<br>Murnane, Jonathan; University of Edinburgh, Queens Medical Research Institute and Edinburgh Imaging facility (QMRI)<br>Whalley, Heather; University of Edinburgh, Centre for Clinical Brain Sciences Centre for Genomic and Experimental Medicine<br>Pernet, Cyril; University of Edinburgh, Queens Medical Research Institute and Edinburgh Imaging facility (QMRI)<br>Horne, Andrew
**Primary Subject Heading**:	Obstetrics and gynaecology
Secondary Subject Heading:	Pharmacology and therapeutics
Keywords:	Chronic pelvic pain, Functional Magnetic Resonance Imaging, Pilot Randomised Control Trial (RCT), Gabapentin, Placebo

SCHOLARONE™
Manuscripts

The use of brain functional magnetic resonance imaging to determine the mechanism of action of gabapentin in managing chronic pelvic pain in women: a pilot study

Authors

Marta Seretny MD, MPH, PhD, FRCA

Univeristy of Edinburgh, Cancer Research UK Centre

Sarah Murray MBChB, MRCOG

University of Edinburgh, MRC Centre for Reprodutive Health

Lucy Whitaker MBChB, MSc, MRCOG, MD

University of Edinburgh, MRC Centre for Reprodutive Health

Jonathan Murnane BSc, MSc

University of Edinburgh, Imaging Facility (QMRI)

Heather C Whalley University of Edinburgh,

Centre for Brain Sciences

Cyril R Pernet PhD

University of Edinburgh, Imaging Facility (QMRI)

*Andrew W Horne MB ChB, PhD, FRCOG

University of Edinburgh, MRC Centre for Reprodutive Health

*Contact Details Corresponding Author:

Prof Andrew Horne

Email: Andrew.Horne@ed.ac.uk

For peer review only

Address: MRC Centre for Reproductive Health, The University of Edinburgh

Queen's Medical Research Institute

47 Little France Crescent

Edinburgh EH16 4TJ

Phone: +44 131 242 6162

Word Count: 2549

For peer review only

Article Summary

Strengths and Limitations of this study:

- This pilot study includes women with chronic pelvic pain (CPP) randomised to receive placebo or gabapentin for its management.
- All participants in this pilot study underwent a brain fMRI scan.
- This study estimated sample size for a larger fMRI study assessing gabapentin in CPP and also assessed study acceptability.
- The fMRI sample was too small to draw any statistical conclusions, all results are suggestive of trends only.

Key Words:

Chronic Pelvic Pain, Functional Magnetic Resonance Imaging (fMRI), Gabapentin, Placebo, Pilot Randomised Control Trial (RCT), Pilot Study

For peer review only

Abstract

Objective: To inform feasibility and design of a future randomised controlled trial (RCT) using brain functional magnetic resonance imaging (fMRI) to determine the mechanism of action of gabapentin in managing chronic pelvic pain (CPP) in women.

Design: Mechanistic study embedded in pilot RCT.

Setting: University Hospital

Participants: Twelve women (18–50 years) with CPP and no pelvic pathology (follow up completed March 2014).

Intervention: Oral gabapentin (300-2700mg) or matched placebo.

Outcome measures: After 12 weeks of treatment, participants underwent functional MRI (fMRI) of the brain (Verio Siemens 3T MRI) during which noxious heat and punctate stimuli were delivered to the pelvis and arm. Outcome measures included pain (visual analogue scale), Blood Oxygen Level Dependent (BOLD) signal change and a semi structured acceptability questionnaire at study completion prior to unblinding.

Results: Full datasets were obtained for 11 participants. Following noxious heat to the abdomen the gabapentin group (GG) had lower pain scores (Mean: 3.8 [SD 2.2]) than the placebo group (PG) (Mean: 5.8 [SD 0.9]). This was also the case for noxious heat to the arm with the GG having lower pain scores (Mean: 2.6 [SD 2.5]) then the PG (Mean: 6.2 [SD 1.1]). Seven out of 12 participants completed the acceptability questionnaire. 71% (five out of seven) described their participation in the fMRI study as positive; the remaining two rated it as a negative experience.

Conclusions: Incorporating brain fMRI in a future RCT to determine the mechanism of action of gabapentin in managing CPP in women was feasible and acceptable to most women.

For peer review only

Trial Registration: ISRCTN70960777.

For peer review only

Introduction

Chronic pelvic pain (CPP) affects >3.3 billion women worldwide and costs an estimated >$225 million/year (£150 million) in socioeconomic costs in the UK alone[1]. Underlying pathology, such as endometriosis or adhesions, can be identified in around 65% of women with CPP. In the remaining 35% of women, pain symptoms have no clear aetiology[2 3]. It is increasingly recognised that women with CPP of unknown aetiology likely represent a subgroup of patients who have undergone central sensitisation (CS), an increased responsiveness of central nociceptive neurons to their normal or sub-threshold afferent input[4]. The treatment for women with CPP of unknown aetiology is challenging[5].

Currently there are no evidence-based analgesics for CPP[6] but gabapentin (a GABA analogue, eliciting its analgesic effects via the spinal N-type calcium channel[7]) is increasingly prescribed due to its known effectiveness in other neuropathic pain states[6]. The true efficacy, mechanism of action and side effect profile of gabapentin in women with CPP remains unknown. However, in general investigation of analgesic drug efficacy in clinical trials is not straightforward. Due to the subjective nature of pain and the influence of an active physiological placebo response, trials investigating analgesics, even when appropriately powered and carefully executed, often produce small effect sizes that are difficult to interpret clinically [8]. Nevertheless, the increase in the use of adjuvant research tools, such as brain functional magnetic resonance imaging (fMRI), has been shown to strengthen and mechanistically explain findings[9].

We therefore undertook a pilot exploratory study to determine the feasibility of investigating the mechanism of action of gabapentin in managing women with CPP using brain fMRI. The objectives of the study were to:

For peer review only

1) To determine whether it is possible to recruit women with CPP, who had agreed to participate in a related pilot clinical trial and received gabapentin or placebo, to partake in an embedded mechanistic study and undergo a brain fMRI scan.

2) To analyze the fMRI brain data and use it for sample size calculation for a future large fMRI brain study investigating the mechanism of action of gabapentin in managing CPP in women.

3) To assess if the fMRI and associated noxious stimuli were acceptable to participants.

For peer review only

Materials and Methods

Study design

This was a pilot exploratory mechanistic study. The flow of patients in the study is detailed in a CONSORT diagram (see figure 1).

Setting

Women with CPP in NHS Lothian (UK), already recruited as part of a pilot RCT conducted in NHS Lothian and NHS Grampian (UK) to investigate the efficacy of gabapentin versus placebo in CPP management[10 11], were approached and invited to participate in this embedded fMRI substudy. The pilot RCT aimed to enrol 60 participants over a nine-month period. The brain fMRI scans were carried out on a Siemens Verio 3T MRI Scanner located at the Edinburgh Imaging Facility, Queens Medical Research Institute (previously known as the Edinburgh Clinical Research Imaging Centre), at the University of Edinburgh (UK). A 12 channel receiver head coil was used. Other study measures were tested in NHS Lothian clinical research facilities or, where appropriate, via the telephone.

For peer review only

Inclusion criteria

- Women aged between 18 and 50 years
- Chronic pelvic pain (non-cyclical pain with or without dysmenorrhoea or dyspareunia) assessed clinically to be located within the true pelvis or between and below anterior iliac crests[12]
- Associated functional disability
- No obvious pelvic pathology at laparoscopy (<36 months and >2 weeks ago)
- Normal pelvic ultrasound (<36 months)

- Using effective contraception e.g. barrier methods, combined oral contraceptive pill, etc.
- Written informed consent

Exclusion criteria

- Known pelvic pathology (for example, endometriosis, cyst, fibroid>3cm)
- Past history of having taken gabapentin or pregabalin
- Due to undergo surgery in the next six months
- History of significant renal impairment
- Allergic to gabapentin or excipients
- Breast feeding
- Pregnancy or planning pregnancy in the next six months
- Metal implant/pacemaker/claustrophobia (fMRI subgroup).

Intervention

Patients were randomised to receive gabapentin at an increasing dose (300-2700mg) or identical placebo tablet. Gabapentin was started at 300mg daily and titrated up weekly, in 300mg increments or until the patient perceived a 50% reduction in pain, or had side effects, to a maximum of 2700mg. Full details are published in the related clinical trial protocol and results[10 11].

Outcome Measures

For peer review only

Women underwent an fMRI scan of the brain after a minimum of twelve weeks of treatment, to ensurine that all participants had reached their steady state dosing regimen at the time of scanning. The following sequences were collected: structural MPRAGE sequence, resting state BOLD sequence where participants were asked to fixate on a cross, BOLD sequences during which punctate and noxious heat stimuli were applied to the arm and lower abdomen respectively. Full details of MRI acquisition parameters are shown in table 1. The order of the presented stimuli was randomised (some participants had thermal stimulation applied to the arm then abdomen, whilst others had it applied in the reverse order). Thermal stimulus was individually thresholded outside the scanner for each participant. For the arm, a *MEDOC Pathway* thermode was placed on the inner arm, three fingerbreadths proximal to the wrist joint. Temperature was increased from 30C until the patient reported a pain score of five out of ten. The same was repeated for the lower abdomen (three finger breadths above the pubic bone) until the patient reported a pain score of five out of ten. These temperatures were used in the scanner in a block experimental design; temperature at neutral for 10seconds and then increased to the participant's pre-established noxious heat threshold and held for 15seconds and then returned to neutral). The duration of the thermal blocks was ten minutes on the arm and ten minutes on the lower abdomen. Pain scores were recorded in real time during the application of the thermal stimulus using a visual analogue scale presented to participants on a screen inside the scanner. Each participant used an automated pointer to rate her pain. Standardised punctate stimuli were applied to the lower abdomen, three fingerbreadths above the pubic bone using a 300g *Touch Test*^{TM} von Frey filament (6.65mm) in an event related experimental design. The full duration of the punctate experiment was ten minutes.

**Weighting**	**T1 weighted image**	**Functional MRI (T2*)**
Pulse sequence	Inversion Recovery	Fast Gradient echo

Imaging type	MPRAGE	Echo Plannar Imaging
Flip angle	9	90
Echo time (TE)	2.98 ms	30 ms
Repetition time (TR)	2.3 sec	2.5 sec
Number of volumes	n/a	Thermal runs: 245<br>Punctate run: 260<br>Resting state: 200
Number of discarded volumes	n/a	0
Phase encoding	Anterior / Posterior	Anterior / Posterior
Parallel imaging	GRAPPA *2	GRAPPA *2
Slice order and timing	Interleaved	Interleaved
Slice position procedure	ACPC	ACPC
Brain Coverage	Full brain	Full brain
Field of View	256	192
Matrix size	256 * 256	64*64
Slice Thickness	1 mm	3
Prospective Motion correction	None	On

Table 1. MRI acquisition parameters. Details provided follow recommendations of the COBIDAS report (http://biorxiv.org/content/early/2016/07/10/054262)

For peer review only

To assess the acceptability of the fMRI substudy, participating women were invited to complete a semi-structured questionnaire administered by telephone at the conclusion of the study protocol prior to unblinding. The questions were designed to assess the participant's feelings and experiences related to the fMRI scan (table 2). The responses were recorded on a structured coding sheet. Respondents were asked to answer each question using a five-point positive (one) to negative (five) scale, with the option of adding free comments.

**Regarding the fMRI substudy**	**Positive**				**Negative**
	1	2	3	4	5
A. … was the choice to opt-in or out a positive or negative one? Did you feel pressured?					
B. … did you feel you were fully informed as to why you were having it?					
C. … was the fMRI a comfortable experience?					
D. … how did you find having the blood sample taken?					
E. … experiences and thoughts on the pain threshold exam					

Table 2. Semi Structured Study Acceptability Questionnaire. Women were asked these questions during a phone interview-taking place prior to study unblinding. The researcher recorded free comments elaborating each question.

Statistical Analysis

All statistical analysis was carried out prior to participant and clinician unblinding. FMRI statistical analysis was carried out using *Statistical Parametric Mapping* (SPM12) software[13]. Functional MRI data first underwent a slice timing correction (interpolation based on a fast Fourier transform FFT, 45th slice as reference), followed by motion correction (six degrees affine registration minimizing the mean square difference, realigned to the first scan of each

For peer review only - http://bmjopen.bmj.com/site/about/guidelines.xhtml

session then to the mean, fourth degree B-spline interpolation). The T1 weighted image was enhanced using SUSAN[14] from *FMRIB Software Library* (FSL). Images were then coregistred to the mean EPI image (12 degrees affine transformation maximizing the normalized mutual information with no interpolation applied) and then simultaneously segmented/bias corrected to derive normalization parameters to the MNI space[15]. Volume based inter-subject registration was next performed applying the normalization parameters to all EPI images (fourth degree B-spline interpolation, final voxel size [2 2 2]). These images were finally smoothed with a 6mm isotropic Gaussian kernel. For the first level analysis the stimuli of each session were modelled convolving stimulus onsets with a double gamma haemodynamic response function with derivates[16] and durations of zero seconds. Design matrices also included standard six motion parameters and filters for low frequency (high pass filter 128 seconds) and high frequency noise (first order auto-regressive plus Gaussian whitening). For the thermal stimuli, presentation scales were also modelled for design completeness but not investigated. Regions of interest (ROIs) were defined using NeuroSynth[17] with the keywords chronic pain (http://neurosynth.org/analyses/terms/chronic%20pain/). The mean beta values among voxels in 6mm spheres centred on the prefrontal thalamus ([+/-10 -15 4]), the insulae (Iq2 [+/-37 -20 14]), the peri-acqueducal gray (PAG - [0 23 -17]) and whole brain stem ([0 -39 54]). These were obtained for each subject and group comparison was performed for each ROI (percentile bootstrap on median difference, p=0.05 Bonferroni corrected – Wilcox, 2015). Power calculations to calculate the sample size needed for 80% power at a significance level of p=0.05, were conducted using the *GPower 3.1* program[18]. The entire dataset was prepared following the Brain Imaging Data Structure (BIDS)[19] and is available on the University of Edinburgh DataShare repository (Horne et al., 2018 https://doi.org/10.7488/ds/2411). Statistical maps are available on NeuroVault[20] (https://neurovault.org/collections/5029/).

Qualitative Analysis

Questionnaire data was assessed thematically in order to identify participant's feelings and experiences related to the fMRI scan and associated noxious heat thresholding. Free comments were reviewed to gain greater understanding on themes important to individual women in the study.

Ethical Approval

The Scotland A Research Ethics committee gave approval for this study (REC 12/SS/0005) on November 13th 2012. All participants gave written informed consent prior to taking part in this study. Consent was also reconfirmed verbally before every fMRI scan.

Patient and Public Involvement

The patient co-founder of the Pelvic Pain Support Network helped with the design of this study and also assisted with gathering feedback regarding the proposed study within the charity. A short description of the proposed pilot study, planned resulting RCT and a series of related questions were distributed amongst members. Encouragingly, from a survey of 85 women involved in the charity, 69% responds said that they would consider taking part in this pilot.

Results

Recruitment

137 women fulfilled the eligibility criteria for the main pilot clinical trial, and 47 women were randomised to treatment[11]. Of the 47 women who were randomised, Women taking part in the RCT at the Edinburgh University Hospital were offered participation in the fMRI sub-study. 12 consecutive participants were approached to take part in the fMRI sub-study. All of

For peer review only

these women consented to undergo a brain fMRI scan, completing the full scanning protocol. Their baseline demographics are shown in table 3. One subject (on placebo) was excluded from analysis following discovery of corrupted scan data at the analysis stage of the study. Progression of pain ratings throughout the study, maximum dose of gabapentin achieved, time to reach maximum dose and reported side effects are summarised in table 4.

For peer review only

**Characteristic**	**Gabapentin (% of 6 or 95% CI)**	**Placebo (% of 6 or 95% CI)**
Age (years)	31.7 (20.3 – 43.0)	30.3 (23.1 – 37.5)
Pain Score (0-10)	6.6 (5.8 – 7.5)	5.5 (3.3– 7.7)
BMI (kg/m^2)	25.05 (21.0 – 29.1)	26.05 (21.2– 30.9)
HADS score (max score 21)	9.7 (6.6 – 12.7)	8 (3.4 -12.5)
Parity:		

Nulliparous<br>Parous	4 (66.7%)<br>2 (33.3%)	4(66.7%))<br>2(33.3%))
Higher Education Received:<br>Highschool<br>College/University	 0(0%)<br>6(100%)	 1(16.7%)<br>5(83.3%)

Table 3. Demographics of women who underwent fMRI brain. HADS= hospital anxiety and depression score.

ID	Group	Baseline Pain	3mo Pain	6mo Pain	Max Dose (mg)	Time to Max Dose	Side effects noted
0104	Active	7	4	3	1800	6	Dry mouth, itchy skin
0107	Active	7	6	4	2100	6	Tired
0113	Placebo	6	6	4	600	8	Tired, dizzy, nightmares
0114	Placebo	6	4	6	2400	8	Dizzy, nausea
0116	Placebo	7	4	5	2400	12	Dizzy, nausea
0119	Placebo	5	4	6	300	*	Dizzy, nausea
0120	Placebo	5	2	3	600	1	Tired, dizzy
0122	Placebo	2	1	2	2100	15	None
0123	Active	8	7	6	2400	12	Restlessness
0124	Placebo	8	7	5	1800	12	Low mood, nausea
0125	Active	6	3	2	1800	8	Dizzy, nausea
0129	Active	6	4	3	1800	7	None

For peer review only

Table 4. Showing pain score (0-10 scale) changes during treatment, maximum treatment dose as well as reported side effects. ID= anonymised study ID number. Active= gabapentin recipients. Time to max dose in weeks. *=missing data

fMRI Analysis

fMRI brain scan data for six subjects receiving gabapentin and five receiving placebo was analysed. Average pain ratings during the fMRI scan following noxious stimuli of the abdomen were lower for the gabapentin group (Mean: 3.8 [SD 2.2]) than the placebo group (Mean: 5.8 [SD 0.9]). This was also the case for noxious heat applied to the arm where the gabapentin group had lower average pain scores (Mean: 2.6 [SD 2.5]) than the placebo group (Mean: 6.2 [SD 1.1]). Figure 2 available at https://drive.google.com/open?id=1Iroa8uxXLQ-w6-kBPbVtqPagLjTjtuPu shows unthresholded activation maps for each of the presented stimuli as well as ROIs and the statistical comparison between these. To summarise, the ROI analysis showed BOLD signal changes in the left PI following punctate stimuli, was significantly different between those receiving placebo as compared to those receiving gabapentin (figure 2), this was also the case for activation in the PAG following heat application to the lower abdomen. All other ROI comparisons showed no statistically significant differences (tables 5,6,7). As expected, due to the exploratory sample size, group level whole brain analysis did not yield any statistically significant between group differences.

**Region**	**Gabapentin**	**Placebo**	**Difference**	**P value**
Left Insula	-0.14	-0.23	[-0.98 0.82]	0.8820

	[-1.71 0.44]	[-1.01 0.14]		
Right Insula	-0.38<br>[-0.52 0.01]	-0.19<br>[-0.51 0.13]	[-0.54 0.32]	0.5160
Left Thalamus	0.97<br>[-0.28 2.44]	1.97<br>[-1.12 3.31]	[-2.53 1.4]	0.3620
Right Thalamus	-0.11<br>[-0.83 0.03]	-0.08<br>[-0.37 0.66]	[-0.84 0.23]	0.7360
PAG	-0.35<br>[-0.7 -0.004]	0.0004<br>[-0.07 0.09]	[-0.61 -0.05]	**0.004**
Brain Stem	-0.3579<br>[-1.25 0.21]	-0.52<br>[-3.71 0.61]	[-1 2.5]	0.7900

Table 5. ROI median beta values with 95% highest density intervals following thermal stimulation to the abdomen. Please note difference = percentile bootstrap median difference and associated p value of this.

Region	Gabapentin	Placebo	Difference	P value
Left Insula	0.01<br>[-1 0.52]	0.19<br>[-0.07 0.4]	[-0.95 0.25]	0.454
Right Insula	-0.13<br>[-0.33 0.1]	0.14<br>[-0.42 0.39]	[-0.55 0.22]	0.254
Left Thalamus	0.53<br>[-0.44 0.88]	1.68<br>[-0.2 2.34]	[-2.01 0.1]	0.07
Right Thalamus	-0.09<br>[-1.03 0.4]	0.13<br>[-0.01 0.3]	[-0.91 0.21]	0.388
PAG	-0.11<br>[-0.42 0.1]	-0.05<br>[-0.5 0.29]	[-0.42 0.36]	0.84
Brain Stem	0.5	1.8	[-2.23 0.28]	0.096

For peer review only

	[-0.42 1.16]	[-0.52 2.72]		

Table 6. ROI median beta values with 95% highest density intervals following thermal stimulation to the hand. Please note difference = percentile bootstrap median difference and associated p value of this.

Region	Gabapentin	Placebo	Difference	P value
Left Insula	0.14<br>[-0.35 0.54]	-0.33<br>[-1 -0.1]	[0.1 1.07]	**0.042**
Right Insula	-0.24<br>[-0.71 0.26]	0.25<br>[-0.65 -0.018]	[-0.3 0.6]	0.892
Left Thalamus	-0.95<br>[-2.5 -0.14]	-0.34<br>[-1.28 1.99]	[-3.45 0.2]	0.114
Right Thalamus	-0.11<br>[-1.99 0.72]	-0.09<br>[-0.14 -0.06]	[-1.1 0.7]	0.948
PAG	-0.05<br>[-0.45 0.16]	-0.21<br>[-1.16 0.39]	[-0.4 0.99]	0.654
Brain Stem	-0.54<br>[-1.59 1.01]	0.12<br>[-4.63 1.62]	[-2.1 2.49]	0.65

Table 7. ROI median beta values with 95% highest density intervals following punctate stimulation to the lower abdomen. Please note difference = percentile bootstrap median difference and associated p value of this.

A sample size calculation from these results, suggest that for a mechanistic sub study embedded in a larger double blind RCT investigating the efficacy of gabapentin versus placebo in CPP, a minimum of 7 women per group (a total of 14 brain fMRI scans) are required for 80% power and p=0.05 significance level, sampling ratio 1:1, with two independent samples and two-sided testing. This calculation was based on differences in

For peer review only

BOLD signal activation in the left insula (punctate) and PAG (abdomen thermal stimuli) or if averaging across all ROI and all conditions.

FMRI Substudy Acceptability

All twelve women were approached for the telephone interview. Seven were contactable and gave consent to the telephone administered semi- structured acceptability questionnaire. Five women rated the fMRI as a positive experience, scoring it as 1 or 2 on a 1 - 5 point, positive to negative scale. Two participants scored their experience as 'four' on the scale. One of these commented that the trial substudy process was not explained clearly enough. This participant was also disappointed by lack of feedback after the scan. The other participant rating the experience negatively felt that an in-person explanation rather than the information leaflet would have been clearer. All participants felt the option of opting in or out of the mechanistic fMRI substudy was positive. The majority of women found the experience of the actual scan positive or neutral, with similar ratings given to the quality of information given before the scan, as well as their experience of the noxious pain thresholding. One participant noted that prior to the study she had anxiety related to MRI scans and that study participation actually helped her overcome her fear of MRI. Only one participant felt that the information given before the scan, the actual scan experience, as well as the noxious thresholding was a negative experience for her (a score of four on the five-point scale).

Discussion

Recruitment

Our study met our objectives in relation to establishing feasibility of recruitment. All of the participants approached for brain fMRI consented to this part of the study and completed

For peer review only

the full hour-long protocol in the scanner. Failure to recruit to trials is known to be a major limitation in clinical research, prompting new recommendations aimed at improving approaches to recruitment[21]. Assessment of study feasibility is a part of this improvement process. Pilot studies are considered a subtype of formal feasibility assessments[22]. Further, pilot studies are deemed valuable in ascertaining unknown factors such as recruitment to novel outcome measures, such as in our case brain fMRI in CPP patients[23]. We feel our successful recruitment to this study supports future fMRI research investigating the mechanism of action of gabapentin for the management of women with CPP.

fMRI Analysis

The key outcome of our fMRI data analysis was calculation of sample size for a planned future brain fMRI study to investigate the mechanism of action of gabapentin in the management of women with CPP. Sample size calculations for fMRI studies have become a standard part of good MRI research methodology[24]. We also explored BOLD signal changes in response to punctate and noxious heat stimuli, in regions of the brain known to be involved in pain processing. Our findings suggest between group differences in response to punctate and abdominal heat stimuli in the insula and PAG. These trends fit with what is known about the fundamental role of the insula and the PAG in central sensitisation and pain processing[25 26]. Specifically, in CPP both the PAG and insula are known to have altered structure and connectivity[27 28 29]. Moreover, central sensitisation is postulated to be an important mechanism in the maintenance of CPP[30 31]. Neuromodulators such as gabapentin have been shown in neuroimaging studies to modify central sensitisation pain states[32]. Further work in a larger mechanistic fMRI study is needed to confirm and elucidate these findings.

Study Acceptability

For peer review only

Patient engagement in clinical trials is known to improve study recruitment and retention[33]. Specific to pain research and analgesic RCTs, many issues limit patient participation in pain trials utilising fMRI, not least the actual experience of chronic pain[34]. Decreased participation of subsets of pain patients in pain medication trials can introduce potential biases into study findings [35]. Understanding patient experiences, which in our case helped highlight the need for clearer communication around study logistics, will help utilise fMRI in future mechanistic probing of gabapentin efficacy in CPP. This is particularly valuable, as the utility of fMRI in chronic pain management research is deemed to have growing importance[36].

Study Strengths and Limitations

The major limitation of our fMRI data analysis lies in our small sample size. Approaches to brain fMRI analysis are varied and maybe prone to false positive or non-reproducible results[37]. This is particularly true when small datasets are used [38]. It is therefore possible that a different approach to the analysis of our data may have yielded different results. We therefore interpret these findings as interesting trends, in need of further validation, and we have made our fMRI dataset available to interested researchers who may wish to re analyse our data or combine it with another relevant dataset. Another limitation of our study is lack of data regarding the duration of CPP in study participants, we have corrected this in the ongoing GaPP2 study. The strengths of this study relate to all the study aims being fulfilled and clearly reported. This study is one of the first to utilise fMRI in the assessment of women with CPP receiving gabapentin or placebo.

Conclusion

For peer review only

We have demonstrated that it is possible to recruit women with CPP participating in a pilot RCT of gabapentin versus placebo, to take part in a mechanistic substudy involving a brain fMRI scan. Most participants in our pilot found the MRI scan, including administration of noxious stimuli acceptable. BOLD signal changes identified in our analysis are likely useful guides for further studies aimed at understanding the mechanism of action of gabapentin in CPP. Sample size calculations resulting from this work have been used to inform the design of a mechanistic fMRI substudy in a larger RCT investigating gabapentin versus placebo for the management of CPP in women.

For peer review only

Figure Legends

Figure 1. CONSORT diagram

Figure 2. Activation maps (unthresholded) for each stimulus presented, alongside ROI group comparisons. Available at https://drive.google.com/file/d/1Iroa8uxXLQ-w6-kBPbVtqPagLjTjtuPu/view

For peer review only

Conflict of Interest

We have read and understood the BMJ Open policy on declaration of interests and declare that we have no competing interests

Author Statement

MS, AH, CP where involved in the design and set up of the study. MS, LW, SM, JM and CP were involved in scan data collection. CP analysed the fMRI data. CP and HW performed the sample size calculation. All authors contributed to the writing of this manuscript.

Data Statement

All data from this study is currently held securely at the University of Edinburgh. Anonymised fMRI data is available to interested researchers and can be accessed at: https://datashare.is.ed.ac.uk/handle/10283/3153

Acknowledgments

We thank Katy Vincent (DPhil, BSc, MBBS, MRCOG) who was instrumental in guiding the set up and data analysis for this study. Thank you to Scott Semple (PhD) and Neil Roberts (PhD), MRI physicists for their assistance in setting up additional data acquisition equipment and monitoring in the MRI scanner. We would also like to thank Ms Jennifer Brawn (MSc, DPhil) for organising scan sequence transfer and set up between Oxford and Edinburgh. We thank all the radiographers at the Clinical Research Imaging Centre (CRIC) Edinburgh, for their contribution to data collection. We also thank Mrs Ann Doust and Mrs Helen Dewart for organising patient recruitment and follow up. Additional thanks are owed to Ann Doust for managing all study data and answering endless queries related to writing this manuscript.

For peer review only

We would like to thank Judy Birch JB, the patient liaison and representative of the Pelvic Pain Support Network for her advice and input into this study. Finally, and perhaps most importantly we would like to thank all the women who took part in this study.

Funding

This work was supported by a grant from the Chief Scientist's Office Scotland (CZH/4/688) and an MRC Centre Grant Mr/N022556/1. MS was funded by the Wellcome Trust via the Scottish Translational Medicine and Therapeutics Initiative (STMTI). JM held a PhD studentship funded by the Mentholatum company. The funders did not have any influence on the study design, data collection, analysis or interpretation of results. The authors do not have any conflict of interest.

For peer review only

References

1. Ahangari A. Prevalence of Chronic Pelvic Pain Among Women: An Updated Review. *Pain Physician* 2014;17(2):E141-E47.

2. Daniels JP, Khan KS. Chronic pelvic pain in women. *BMJ* 2010;341 doi: 10.1136/bmj.c4834

3. Howard FM. The role of laparoscopy in chronic pelvic pain: promise and pitfalls. *Obstetrical & gynecological survey* 1993;48(6):357-87. [published Online First: 1993/06/01]

4. Kaya S, Hermans L, Willems T, et al. Central sensitization in urogynecological chronic pelvic pain: a systematic literature review. *Pain Physician* 2013;16(4):291-308. [published Online First: 2013/07/24]

5. Eller-Smith OC, Nicol AL, Christianson JA. Potential Mechanisms Underlying Centralized Pain and Emerging Therapeutic Interventions. *Frontiers in Cellular Neuroscience* 2018;12(35) doi: 10.3389/fncel.2018.00035

6. Royal College of Obstetricians and Gynaecologists. Therapies Targeting the Nervous System for Chronic Pelvic Pain Relief. 2015 [Available from: https://www.rcog.org.uk/globalassets/documents/guidelines/scientific-impact-papers/sip46.pdf2015.

7. Cheng JK, Chiou LC. Mechanisms of the antinociceptive action of gabapentin. *Journal of pharmacological sciences* 2006;100(5):471-86. [published Online First: 2006/02/14]

8. Dworkin RH, Peirce-Sandner S, Turk DC, et al. Outcome measures in placebo-controlled trials of osteoarthritis: responsiveness to treatment effects in the REPORT database. *Osteoarthr Cartilage* 2011;19(5):483-92.

9. Wise RG, Tracey I. The role of fMRI in drug discovery. *Journal of Magnetic Resonance Imaging* 2006;23(6):862-76. doi: 10.1002/jmri.20584

10. Horne AW, Critchley HO, Doust A, et al. GaPP: a pilot randomised controlled trial of the efficacy of action of gabapentin for the management of chronic pelvic pain in women: study protocol. *BMJ open* 2012;2(3) doi: 10.1136/bmjopen-2012-001297 [published Online First: 2012/06/12]

11. Lewis SC, Bhattacharya S, Wu O, et al. Gabapentin for the Management of Chronic Pelvic Pain in Women (GaPP1): A Pilot Randomised Controlled Trial. *PloS one* 2016;11(4):e0153037. doi: 10.1371/journal.pone.0153037

For peer review only

12. RCOG Green-top Guideline. The Initial Managment of Chronic Pelvic Pain, 2012:1-16.

13. Flandin G, Friston K. Statistical parametric mapping (SPM). *Scholarpedia,* 2008;3(4):6332. doi: https://doi.org/10.4249/scholarpedia.6232

14. Smith SM, Brady JM. SUSAN - a new approach to low level image processing. . *International Journal of Computer Vision* 1997;23(1):45-78. doi: https://doi.org/10.1023/A:1007963824710

15. Ashburner J, Friston KJ. Unified segmentation. *NeuroImage* 2005;26(3):839-51. doi: 10.1016/j.neuroimage.2005.02.018 [published Online First: 2005/06/16]

16. Friston KJ, Holmes AP, Worsley KJ, et al. Statistical parametric maps in functional imaging: A general linear approach. *Human Brain Mapping* 1994;2(4):189-210. doi: 10.1002/hbm.460020402

17. Yarkoni T, Poldrack RA, Nichols TE, et al. Large-scale automated synthesis of human functional neuroimaging data. *Nat Methods* 2011;8(8):665-70. doi: 10.1038/nmeth.1635 [published Online First: 2011/06/28]

18. Faul F, Erdfelder E, Lang A-G, et al. G*Power 3: A flexible statistical power analysis program for the social, behavioral, and biomedical sciences. *Behavior Research Methods* 2007;39(2):175-91. doi: 10.3758/bf03193146

19. Gorgolewski KJ, Auer T, Calhoun VD, et al. The brain imaging data structure, a format for organizing and describing outputs of neuroimaging experiments. *Scientific data* 2016;3:160044. doi: 10.1038/sdata.2016.44 [published Online First: 2016/06/22]

20. Gorgolewski KJ, Varoquaux G, Rivera G, et al. NeuroVault.org: a web-based repository for collecting and sharing unthresholded statistical maps of the human brain. *Frontiers in neuroinformatics* 2015;9:8. doi: 10.3389/fninf.2015.00008 [published Online First: 2015/04/29]

21. Huang GD, Bull J, Johnston McKee K, et al. Clinical trials recruitment planning: A proposed framework from the Clinical Trials Transformation Initiative. *Contemporary clinical trials* 2018;66:74-79. doi: 10.1016/j.cct.2018.01.003

22. Eldridge SM, Lancaster GA, Campbell MJ, et al. Defining Feasibility and Pilot Studies in Preparation for Randomised Controlled Trials: Development of a Conceptual Framework. *PloS one* 2016;11(3):e0150205. doi: 10.1371/journal.pone.0150205

23. Cooper CL, Whitehead A, Pottrill E, et al. Are pilot trials useful for predicting randomisation and attrition rates in definitive studies: A review of publicly funded trials. *Clinical trials (London, England)* 2018;15(2):189-96. doi: 10.1177/1740774517752113

For peer review only

24. Guo Q, Thabane L, Hall G, et al. A systematic review of the reporting of sample size calculations and corresponding data components in observational functional magnetic resonance imaging studies. *NeuroImage* 2014;86:172-81. doi: 10.1016/j.neuroimage.2013.08.012

25. Segerdahl AR, Mezue M, Okell TW, et al. The dorsal posterior insula subserves a fundamental role in human pain. *Nature neuroscience* 2015;18(4):499-500. doi: 10.1038/nn.3969 [published Online First: 2015/03/10]

26. Lu C, Yang T, Zhao H, et al. Insular Cortex is Critical for the Perception, Modulation, and Chronification of Pain. *Neuroscience bulletin* 2016;32(2):191-201. doi: 10.1007/s12264-016-0016-y

27. Brawn J, Morotti M, Zondervan KT, et al. Central changes associated with chronic pelvic pain and endometriosis. *Human reproduction update* 2014;20(5):737-47. doi: 10.1093/humupd/dmu025

28. As-Sanie S, Harris RE, Napadow V, et al. Changes in regional gray matter volume in women with chronic pelvic pain: A voxel-based morphometry study. *Pain* 2012;153(5):1006-14. doi: Doi 10.1016/J.Pain.2012.01.032

29. As-Sanie S, Kim J, Schmidt-Wilcke T, et al. Functional Connectivity is Associated With Altered Brain Chemistry in Women With Endometriosis-Associated Chronic Pelvic Pain. *J Pain* 2016;17(1):1-13. doi: 10.1016/j.jpain.2015.09.008 [published Online First: 2015/10/13]

30. Grundstrom H, Gerdle B, Alehagen S, et al. Reduced pain thresholds and signs of sensitization in women with persistent pelvic pain and suspected endometriosis. *Acta obstetricia et gynecologica Scandinavica* 2019;98(3):327-36. doi: 10.1111/aogs.13508 [published Online First: 2018/11/26]

31. Levesque A, Riant T, Ploteau S, et al. Clinical Criteria of Central Sensitization in Chronic Pelvic and Perineal Pain (Convergences PP Criteria): Elaboration of a Clinical Evaluation Tool Based on Formal Expert Consensus. *Pain Med* 2018 doi: 10.1093/pm/pny030 [published Online First: 2018/03/10]

32. Iannetti GD, Zambreanu L, Wise RG, et al. Pharmacological modulation of pain-related brain activity during normal and central sensitization states in humans. *Proc Natl Acad Sci U S A* 2005;102(50):18195-200. doi: 10.1073/pnas.0506624102

33. Patrick-Lake B. Patient engagement in clinical trials: The Clinical Trials Transformation Initiative's leadership from theory to practical implementation. *Clinical trials (London, England)* 2018;15(1_suppl):19-22. doi: 10.1177/1740774518755055

34. Moayedi M, Salomons TV, Atlas LY. Pain Neuroimaging in Humans: A Primer for Beginners and Non-Imagers. *J Pain* 2018 doi: 10.1016/j.jpain.2018.03.011

For peer review only

35. Karos K, Alleva JM, Peters ML. Pain, Please: An Investigation of Sampling Bias in Pain Research. *J Pain* 2018 doi: 10.1016/j.jpain.2018.02.011 [published Online First: 2018/03/09]

36. Smith SM, Dworkin RH, Turk DC, et al. The Potential Role of Sensory Testing, Skin Biopsy, and Functional Brain Imaging as Biomarkers in Chronic Pain Clinical Trials: IMMPACT Considerations. *J Pain* 2017;18(7):757-77. doi: 10.1016/j.jpain.2017.02.429

37. Bennett CM, Miller MB. How reliable are the results from functional magnetic resonance imaging? In: Kingstone AMMB, ed. Year in Cognitive Neuroscience2010:133-55.

38. Cremers HR, Wager TD, Yarkoni T. The relation between statistical power and inference in fMRI. *PloS one* 2017;12(11):e0184923. doi: 10.1371/journal.pone.0184923

For peer review only

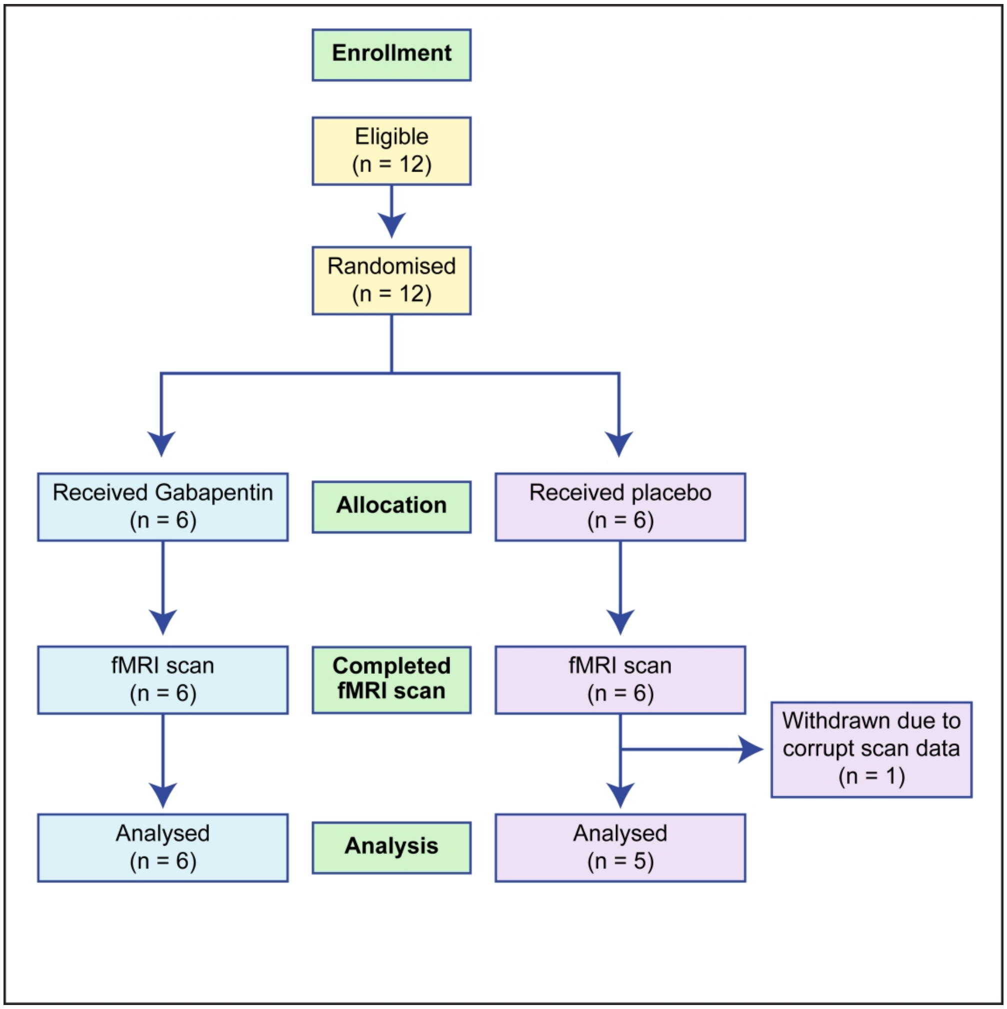

Figure 1. Consort Diagram

149x149mm (600 x 600 DPI)

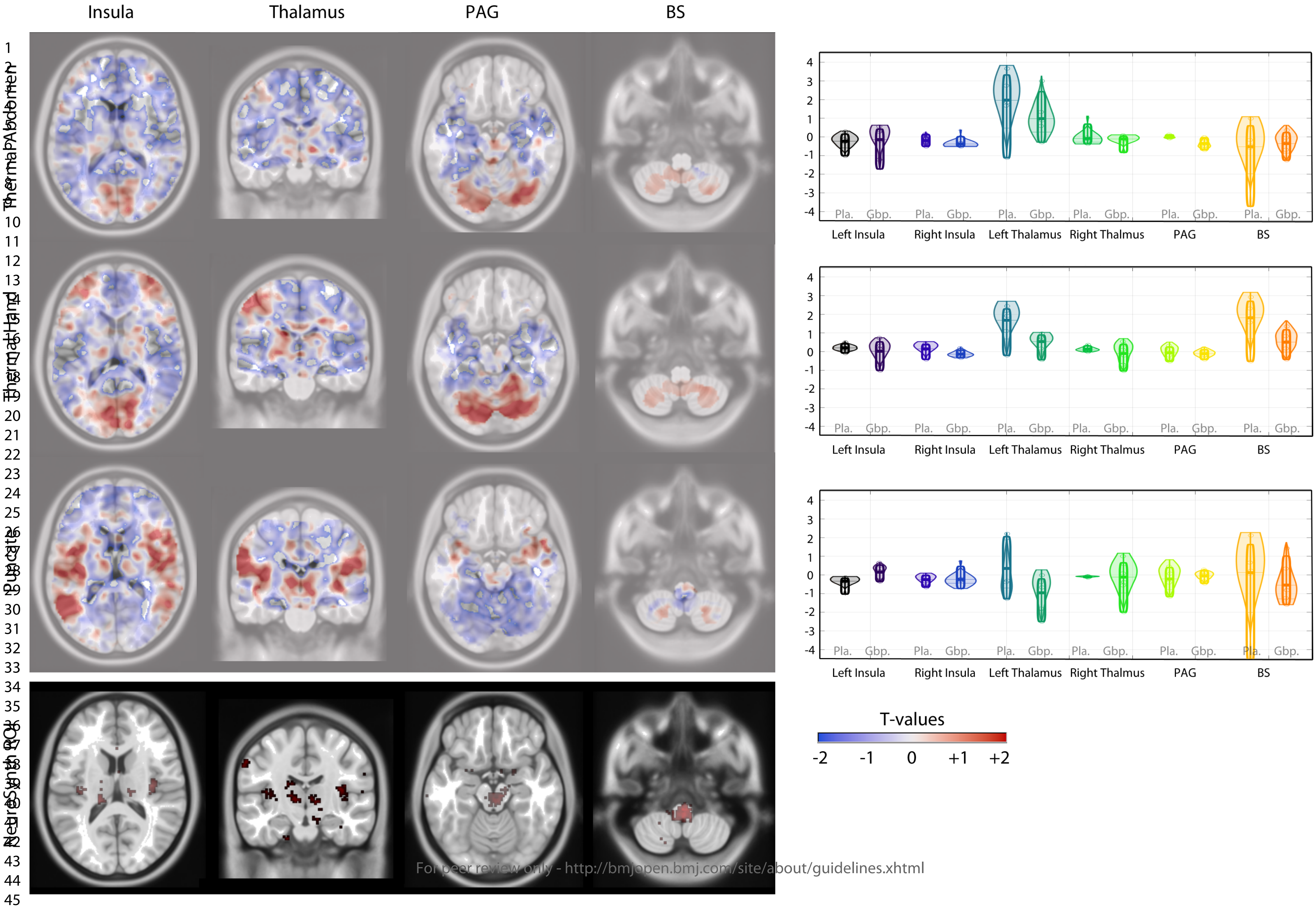

For peer review only - http://bmjopen.bmj.com/site/about/guidelines.xhtml

CONSORT 2010 checklist of information to include when reporting a pilot or feasibility trial*

Section/Topic	Item No	Checklist item	Reported on page No
**Title and abstract**			
	1a	Identification as a pilot or feasibility randomised trial in the title	1
	1b	Structured summary of pilot trial design, methods, results, and conclusions (for specific guidance see CONSORT abstract extension for pilot trials)	3,4
**Introduction**			
Background and objectives	2a	Scientific background and explanation of rationale for future definitive trial, and reasons for randomised pilot trial	5,6
	2b	Specific objectives or research questions for pilot trial	6
**Methods**			
Trial design	3a	Description of pilot trial design (such as parallel, factorial) including allocation ratio	7
	3b	Important changes to methods after pilot trial commencement (such as eligibility criteria), with reasons	n/a
Participants	4a	Eligibility criteria for participants	7,8
	4b	Settings and locations where the data were collected	7
	4c	How participants were identified and consented	7
Interventions	5	The interventions for each group with sufficient details to allow replication, including how and when they were actually administered	8
Outcomes	6a	Completely defined prespecified assessments or measurements to address each pilot trial objective specified in 2b, including how and when they were assessed	9
	6b	Any changes to pilot trial assessments or measurements after the pilot trial commenced, with reasons	n/a
	6c	If applicable, prespecified criteria used to judge whether, or how, to proceed with future definitive trial	11
Sample size	7a	Rationale for numbers in the pilot trial	7
	7b	When applicable, explanation of any interim analyses and stopping guidelines	n/a
Randomisation:			
Sequence generation	8a	Method used to generate the random allocation sequence	8
	8b	Type of randomisation(s); details of any restriction (such as blocking and block size)	8
Allocation concealment mechanism	9	Mechanism used to implement the random allocation sequence (such as sequentially numbered containers), describing any steps taken to conceal the sequence until interventions were assigned	8

For peer review only

Implementation	10	Who generated the random allocation sequence, who enrolled participants, and who assigned participants to interventions	8
Blinding	11a	If done, who was blinded after assignment to interventions (for example, participants, care providers, those assessing outcomes) and how	8
	11b	If relevant, description of the similarity of interventions	8
Statistical methods	12	Methods used to address each pilot trial objective whether qualitative or quantitative	9,10
**Results**			
Participant flow (a diagram is strongly recommended)	13a	For each group, the numbers of participants who were approached and/or assessed for eligibility, randomly assigned, received intended treatment, and were assessed for each objective	14
	13b	For each group, losses and exclusions after randomisation, together with reasons	14
Recruitment	14a	Dates defining the periods of recruitment and follow-up	14
	14b	Why the pilot trial ended or was stopped	14
Baseline data	15	A table showing baseline demographic and clinical characteristics for each group	15
Numbers analysed	16	For each objective, number of participants (denominator) included in each analysis. If relevant, these numbers should be by randomised group	15
Outcomes and estimation	17	For each objective, results including expressions of uncertainty (such as 95% confidence interval) for any estimates. If relevant, these results should be by randomised group	15-19
Ancillary analyses	18	Results of any other analyses performed that could be used to inform the future definitive trial	15-19
Harms	19	All important harms or unintended effects in each group (for specific guidance see CONSORT for harms)	15-19
	19a	If relevant, other important unintended consequences	n/a
**Discussion**			
Limitations	20	Pilot trial limitations, addressing sources of potential bias and remaining uncertainty about feasibility	21
Generalisability	21	Generalisability (applicability) of pilot trial methods and findings to future definitive trial and other studies	21
Interpretation	22	Interpretation consistent with pilot trial objectives and findings, balancing potential benefits and harms, and considering other relevant evidence	21,22
	22a	Implications for progression from pilot to future definitive trial, including any proposed amendments	22
**Other information**			
Registration	23	Registration number for pilot trial and name of trial registry	7,13
Protocol	24	Where the pilot trial protocol can be accessed, if available	7
Funding	25	Sources of funding and other support (such as supply of drugs), role of funders	25
	26	Ethical approval or approval by research review committee, confirmed with reference number	13

For peer review only

Citation: Eldridge SM, Chan CL, Campbell MJ, Bond CM, Hopewell S, Thabane L, et al. CONSORT 2010 statement: extension to randomised pilot and feasibility trials. BMJ. 2016;355.

*We strongly recommend reading this statement in conjunction with the CONSORT 2010, extension to randomised pilot and feasibility trials, Explanation and Elaboration for important clarifications on all the items. If relevant, we also recommend reading CONSORT extensions for cluster randomised trials, non-inferiority and equivalence trials, non-pharmacological treatments, herbal interventions, and pragmatic trials. Additional extensions are forthcoming: for those and for up to date references relevant to this checklist, see www.consort-statement.org.

For peer review only
